# Deciphering the Interactome of *Neisseria meningitidis* With Human Brain Microvascular Endothelial Cells

**DOI:** 10.3389/fmicb.2018.02294

**Published:** 2018-09-26

**Authors:** Evelína Kánová, Irene Jiménez-Munguía, Petra Majerová, Zuzana Tkáčová, Katarína Bhide, Patrícia Mertinková, Lucia Pulzová, Andrej Kováč, Mangesh Bhide

**Affiliations:** ^1^Laboratory of Biomedical Microbiology and Immunology, The University of Veterinary Medicine and Pharmacy in Kosice, Kosice, Slovakia; ^2^Institute of Neuroimmunology of Slovak Academy of Sciences, Bratislava, Slovakia

**Keywords:** *Neisseria meningitidis*, hBMECs, bioinformatic pipeline, BBB, ligands, interactome, outer membrane proteins

## Abstract

*Neisseria meningitidis* is able to translocate the blood-brain barrier and cause meningitis. Bacterial translocation is a crucial step in the onset of neuroinvasion that involves interactions between pathogen surface proteins and host cells receptors. In this study, we applied a systematic workflow to recover and identify proteins of *N. meningitidis* that may interact with human brain microvascular endothelial cells (hBMECs). Biotinylated proteome of *N. meningitidis* was incubated with hBMECs, interacting proteins were recovered by affinity purification and identified by SWATH-MS. Interactome of *N. meningitidis* comprised of 41 potentially surface exposed proteins. These were assigned into groups based on their probability to interact with hBMECs: high priority candidates (21 outer membrane proteins), medium priority candidates (14 inner membrane proteins) and low priority candidates (six secretory proteins). Ontology analysis provided information for 17 out of 41 surface proteins. Based on the series of bioinformatic analyses and literature review, five surface proteins (adhesin MafA1, major outer membrane protein P.IB, putative adhesin/invasion, putative lipoprotein and membrane lipoprotein) were selected and their recombinant forms were produced for experimental validation of interaction with hBMECs by ELISA and immunocytochemistry. All candidates showed interaction with hBMECs. In this study, we present a high-throughput approach to generate a dataset of plausible meningococcal ligands followed by systematic bioinformatic pipeline to categorize the proteins for experimental validation.

## Introduction

*Neisseria meningitidis* (also known as meningococcus) is primarily a human pathogen, which causes meningitis and fatal sepsis. It is normally a commensal in ~10% of the population (Greenfield et al., 1971). *N. meningitidis* colonizes the nasopharynx and can subsequently disseminate into the bloodstream allowing contact with the cells of neurovascular unit, which in turn facilitates translocation of bacteria across the blood-brain barrier (BBB) (Lambotin et al., 2005).

Human BBB tissue is composed of microvascular endothelial cells (hBMECs), pericytes, astrocytes and the basal lamina (Banks, 2015). This structure not only provides protection of the central nervous system (CNS) from microorganisms and toxins but also maintains the brain homeostasis and permits communication between blood and CNS (Banks, 2015). Despite highly sophisticated structure of BBB, some pathogens cross the BBB and invade CNS tissue (Doran et al., 2016).

The interactions between pathogen ligands and receptors on the cells of neurovascular unit initiate various cell signaling events, which may help pathogen to cross BBB. It has been reported that the type IV pili of *N. meningitidis* activate a β2-adrenergic receptor, which leads to the formation of cortical plaques and provides multiple signaling functions that promote bacterial infection (Miller et al., 2013). Opa and Opc surface proteins, located under the polysaccharide capsule, also bind to the endothelial cell receptors (Hardy et al., 2000). However, detailed picture of the ligand-receptor interactors that may take part in the translocation of *N. meningitidis* is still incomplete, which indicates a need of a high-throughput approach to reveal plausible meningococcal interactome.

The study of surface proteins containing highly hydrophobic moieties (e.g., membrane proteins) had been hampered by lack of appropriate technology. Gel-based techniques have been applied for the identification of bacterial membrane proteins as well as their receptors (Pulzova et al., 2011; Bencurova et al., 2015; Mlynarcik et al., 2015), however, these techniques are time-consuming and low-throughput. Moreover, gel-based techniques require a higher amount of biological material, which is an inconvenience in studies where the biological material is scarce (e.g., primary cells of the neurovascular unit). So far, high-throughput genomic approaches have been used to identify genes encoding proteins involved in pathogenicity of *N. meningitidis* (Tettelin et al., 2000; Zhang et al., 2014). This is first study that uses high-throughput mass-spectrometry (MS) to reveal the interactome of N. meningitidis with hBMECs. To identify plausible meningococcal interactome, techniques like labeling of the meningococcal proteome, high-throughput SWATH-MS and bioinformatic pipeline were used here.

Data generated from high-throughput technology requires rigorous data analysis approach to expose relevant biological information and shortlist important gene or protein candidates from large data subset. Bioinformatic analysis applied to a large set of data can help to improve selection of target molecules that are most relevant to the study. We have recently applied a set of bioinformatic tools to select pneumococcal surface proteins that may interact with hBMECs (Jimenez-Munguia et al., 2018). Similar bioinformatic pipeline was set in this study using freely available bioinformatic tools (such as CELLO, Psortb, SignalP, LipoP, HMMtop and TMHMM) to identify bacterial proteins that have less chance to bind host cells (e.g., cytoplasmic proteins) and select interacting proteins (plausible interactome).

In this research, by combining proteome labeling (biotinylation), SWATH-MS and performing bioinformatic analysis we attempted to identify ligands of *N. meningitidis* that may have potential to interact with hBMECs. Bioinformatic analysis was used to select five proteins candidates, which were then expressed in *E. coli* and subsequently shown to interact with hBMECs. Using this approach 41 meningococcal proteins that may interact with hBMECs were detected. We were able to validate interactions of selected bacterial ligands with hBMECs, which may play an important role in adherence.

Results presented in this publication together with our recently published study (Jimenez-Munguia et al., 2018), validate the experimental and bioinformatic approach designed to uncover potential bacterial ligands interacting with the host cells.

## Materials and methods

### *N. meningitidis* culture

*N. meningitidis* (isolate M1/03) was grown on Thayer Martin agar and a single isolated colony was transferred into 50 mL of Brain Heart Infusion Broth (BHI) enriched with 10 mM MgCl_2_. Bacterial culture were grown at 37°C and 5% CO_2_ until OD_600_ = 0.6 (mid-exponential phase). The neuroinvasive *N. meningitidis* isolate used in this study was kindly provided by the University Hospital Olomouc, Czech Republic. Phenotypic characterization (biochemical tests) and genotyping (multilocus sequence typing) was performed in the hospital laboratory.

### Isolation of proteins of meningococci and biotin labeling

Proteins of *N. meningitidis* were extracted exactly as described before (Jimenez-Munguia et al., 2018) in non-denaturating lysis buffer containing 20 mM CHAPS, 300 mM NaCl, 0.01% sodium azide and 1 × proteases inhibitors, and biotinylated with EZ-Link Sulfo-NHS-LC-Biotin (Thermo Fisher Scientific) as per manufacturer's instructions. Biotinylation was confirmed with NeutrAvidin (Thermo Fisher Scientific) capture followed by SDS-PAGE as per manufacturer's instructions. Protein samples were quantified with the Bradford method, aliquoted and stored at −80°C until incubation with hBMECs.

### Human brain microvascular endothelial cell culture

Human BMECs (D3 cell line), were cultured as previously described (Jimenez-Munguia et al., 2018). Cells were either incubated with biotinylated proteins of *N. meningitidis* or scrapped for protein isolation.

### Isolation of proteins from hBMECs

Human BMECs were washed twice with EBM-2 medium (without any supplement) and once with phosphate buffer saline (pH 7.2) to remove albumin. Cells were scraped and resuspended in 1 mL of lysis solution (20 mM CHAPS, 300 mM NaCl, 0.1% sodium azide and 1 × proteases inhibitors). Proteins were extracted as previously described (Jimenez-Munguia et al., 2018) and stored in aliquots at −80°C until use.

### The interaction between biotinylated proteins of *N. meningitidis* and hBMECs

The interaction was performed by incubating 200 μg of biotinylated proteins with confluent hBMECs in 25-mL cell culture flask for 1 h at 37°C in 5% of CO_2_. Cells were washed four times with Dulbecco's-PBS (Sigma) to remove unbound biotinylated proteins. The cell monolayer was then scraped in 2 mL PBS. Cells were centrifuged (3,000 × g, 10 min) and supernatant (S1) was kept on ice until use (please note that S1 may contain biotinylated proteins). The cell pellet was resuspended in 200 μL of lysis solution (cell surface protein isolation kit, Thermo Fisher Scientific) followed by 30 min incubation on ice (every 5 min cells were mixed well by 5 s of vortexing). Supernatant S1 was added to the cells in the lysis solution and five cycles of sonication (100% amplitude, 30 s on ice) were carried out. Proteins-containing supernatant (S2) was collected by centrifugation (10,000 × g, 5 min, 4°C). S2 was kept on ice until the capture of biotinylated proteins.

Presence of the biotinylated proteins in S2 was confirmed by dot blot as described earlier (Jimenez-Munguia et al., 2018).

### Capture of biotinylated proteins from the cell extract (S2) and protein identification

Biotinylated proteins were captured on NeutrAvidin agarose beads (Thermo Fisher Scientific) according to the manufacturer's instructions. In short, NeutrAvidin agarose beads were washed with PBS and incubated with 200 μL of S2 for 1 h. After five wash steps with PBS, biotinylated proteins were eluted in 400 μL of 50 mM dithiothreitol (DTT) in PBS (pH 7.2). Proteins in eluate were identified by mass spectrometry as described in our previous report (Jimenez-Munguia et al., 2018).

### Bioinformatic pipeline: a systematic selection of potential candidates for validation of interaction with hBMECs

**Figure 1:**
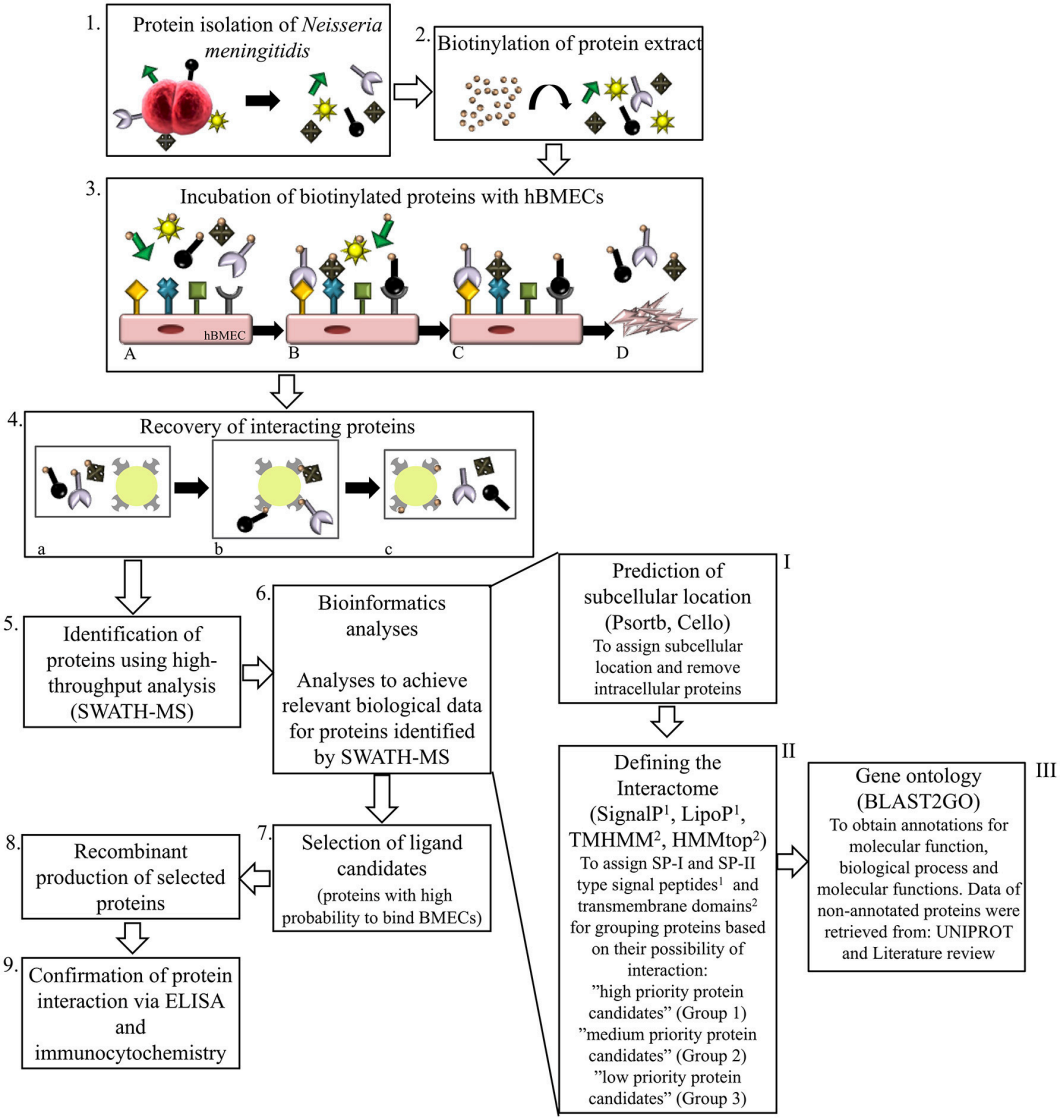
Schematic representation of experimental and bioinformatic workflow performed in this study. Proteins of *N. meningitidis* were isolated^1^ and biotinylated^2^. Biotinylated proteome was incubated with human brain microvascular endothelial cells (hBMECs)^3A^. Unbound proteins were washed off^3Band3C^ and interacting proteins bound to hBMECs were recovered from cell lyste^3D^ with NeutrAvidin capture beads^4aand4b^, eluted with 50 mM DTT^4c^ and identified by SWATH-MS^5^. Combination of bioinformatic tools was used for analyzing the interactome of *N. meningitidis*^6^. Surface proteins were grouped for selection of potential ligands. First analysis^6−I^ (Psortb and Cello) segregated proteins according to subcellular location and intracellular proteins were removed. With the second set of analytic tools^6−II^, interactome was classified into the outer (group 1), inner (group 2) and secretory (group 3) proteins by applying algorithms to predict transmembrane helices (TMHMM and HMMtop) and type I and II signal peptides (SignalP and LipoP). Gene ontology (Blast2GO), UniProt and literature review^6−III^ were performed further on proteins grouped above in 6-II. Bioinformatic pipeline enabled us to select most probable protein candidates that may interact with hBMECs and contribute in pathogenesis^7^. Those protein candidates were overexpressed in *E.coli*^8^ and used for validation with ELISA and immunocytochemistry^9^.

We focused mainly on the cell wall proteins. First, subcellular location was assigned to remove proteins with less probability of interaction (intracellular proteins) and shortlist the surface exposed proteins. Second, surface interactome was classified into three categories according to the probability of interaction with host cells based on subcellular location. Third, ontology annotations were retrieved for surface-exposed proteins (Figure 1).

Primary predictions of subcellular location were assigned with freely available algorithms Psortb (http://www.psort.org/psortb/; Yu et al., 2010), and Cello (http://cello.life.nctu.edu.tw/; Yu et al., 2004). Presence of transmembrane domains was evaluated by TMHMM 2.0 (http://www.cbs.dtu.dk/services/TMHMM-2.0; Krogh et al., 2001) and HMMTOP (http://www.enzim.hu/hmmtop/; Tusnady and Simon, 2001). Presence of type-I and type-II signal peptides were predicted with SignalP (http://www.cbs.dtu.dk/services/SignalP-4.0/; Petersen et al., 2011) and LipoP (http://www.cbs.dtu.dk/services/LipoP; Juncker et al., 2003), respectively. With these predictors, candidates were categorized into the cytoplasmic, membrane-associated (or surface exposed) and secreted proteins.

Gene ontology was then retrieved only for surface-exposed proteins using Blast2GO (https://www.blast2go.com), which provided annotations from characterized proteins, whereas for non-annotated proteins annotations were retrieved from their orthologs (>95% identity). For proteins lacking annotations from Blast2GO, a search was performed in UniProt (http://www.uniprot.org/).

### Synthesis of recombinant forms of the shortlisted proteins

Shortlisted protein candidates were overexpressed in *E. coli*. In brief, gene fragments encoding surface exposed region of proteins were amplified by PCR from genomic DNA of N. meningitidis. Amino acid and nucleotide sequences of amplified regions are presented in Supplementary Data Sheet 1. Detailed information on primer design, amplicon lengths and restriction enzymes used are presented in Supplementary Data Sheet 2. Digestion of amplified PCR products, ligation into a pQE-30-mCherry-GFP plasmid (in-house modified vector pQE-30 UA, Qiagen Comor et al., 2017, transformation and selection of clones were performed as described earlier Jimenez-Munguia et al., 2018. Insertion of encoding genes was confirmed by sequencing with vector specific primers UA Insertom F and R (Supplementary Data Sheet 2). Protein expression and purification with metal affinity chromatography were also carried out as described previously (Jimenez-Munguia et al., 2018). The purity of recombinant proteins was assessed by SDS-PAGE, while molecular mass was measured by MALDI-TOF MS. Protein concentration was measured using Bradford assay and aliquots of purified proteins were stored at −20°C in 20% glycerol until use.

### Confirmation of interaction between recombinant ligands of meningococci and proteins of hBMECs

To confirm the interactions between recombinant ligands of meningococci and proteins of hBMECs an ELISA was performed as described previously (Jimenez-Munguia et al., 2018) with minor modifications. Briefly, wells were coated with protein extract of hBMECs diluted in coating buffer (8 μg of protein in 10 mM Na_2_CO_3_, 40 mM NaHCO_3_, pH 7.2). hBMECs proteins were incubated with various concentrations of each recombinant ligand (10, 20, or 40 μg/mL) for 1 h at room temperature. Bound recombinant proteins were detected with HisProbe-HRP (1 μg/mL dissolved in PBS containing 0.05% Tween-20, 30 min incubation at room temperature, Thermo Fisher Scientific). The reaction was developed with HRP substrate (LI-COR, Biosciences) and measured at 700 nm (Odyssey CLx, LI-COR Bioscience).

For input controls, 2 μg of each recombinant ligand were coated in the wells and detected with HisProbe-HRP. As negative controls, 8 μg of the cell extract of hBMECs were coated in the wells and incubated with HisProbe-HRP. As a positive control, 8 μg of the cell extract of hBMECs were incubated with domain III of protein E of West Nile Virus (known protein that interacts with hBMECs). The assay was performed in triplicates.

### Confirmation of binding of ligands to the cultured hBMECs

Immunocytochemistry was performed as described previously (Jimenez-Munguia et al., 2018) with minor modifications. In brief, hBMECs were cultured on the coverslips coated with collagen type I (Sigma, USA) until 70% confluency. Cells were incubated with purified recombinant ligands (25 μg resuspended in 2 mL EBM-2 medium) for 2 h at 37°C in 5% CO_2_. Cells were washed with PBS containing Tween 20 (0.05%, PBST), fixed with ethanol/acetone (2:8 v/v) for 10 min and washed again three times. Bound recombinant proteins were detected with anti-His antibody conjugated with FITC, whereas nuclei were stained with DAPI (Sigma).

As negative control assay was performed without recombinant ligands. Domain III of protein E of West Nile Virus was also included in the experiment as positive control. Imaging was performed using LSM-710 microscope (Zeiss, Germany). The assay was performed in biological triplicates.

## Results

### Identification of putative surface interactome of *N. meningitidis*

**Figure 2:**
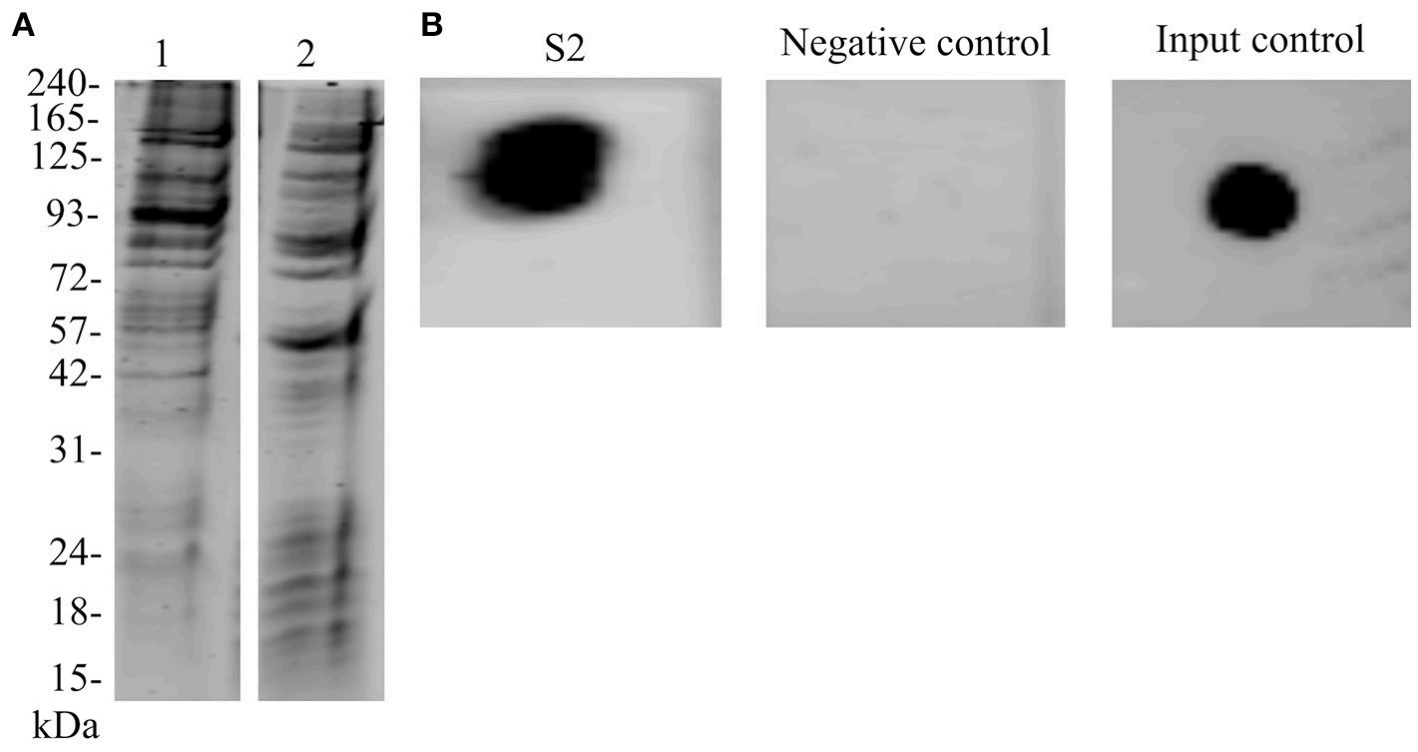
Biotinylation of *N. meningitidis* proteome and confirmation of the presence of biotinylated proteins bound to hBMECs. **(A)** Lane 1–Protein extract of meningococci prior to biotinylation separated on SDS-PAGE. Lane 2–Biotinylated proteins were incubated with NeutrAvidin capture beads, eluted with 50 mM DTT and separated on SDS-PAGE. **(B)** Dot blot. S2–Protein extract of hBMECs obtained after incubation with biotinylated proteins of meningococci spotted on the membrane and detected with IRdye®;800 Streptavidin. Negative control–Total protein extract of hBMECs was spotted on the membrane and incubated with IRdye®;800 Streptavidin, Input control–Biotinylated proteins of meningococci were spotted on the membrane and detected with IRdye®;800 Streptavidin.

Biotinylation of *N. meningitidis* proteome was evaluated by capturing labeled proteins on NeutrAvidin beads. We observed that most of the protein species were recovered when compared with the whole cell extract of *N. meningitidis* (Figure 2A). Recovery of the interacting biotinylated proteins in S2 eluate was evaluated with dot blot (Figure 2B). Further, with the SWATH-MS we succeeded in the identification of 84 proteins, which may bind to the endothelial cells (Supplementary Data Sheet 3).

**Figure 3:**
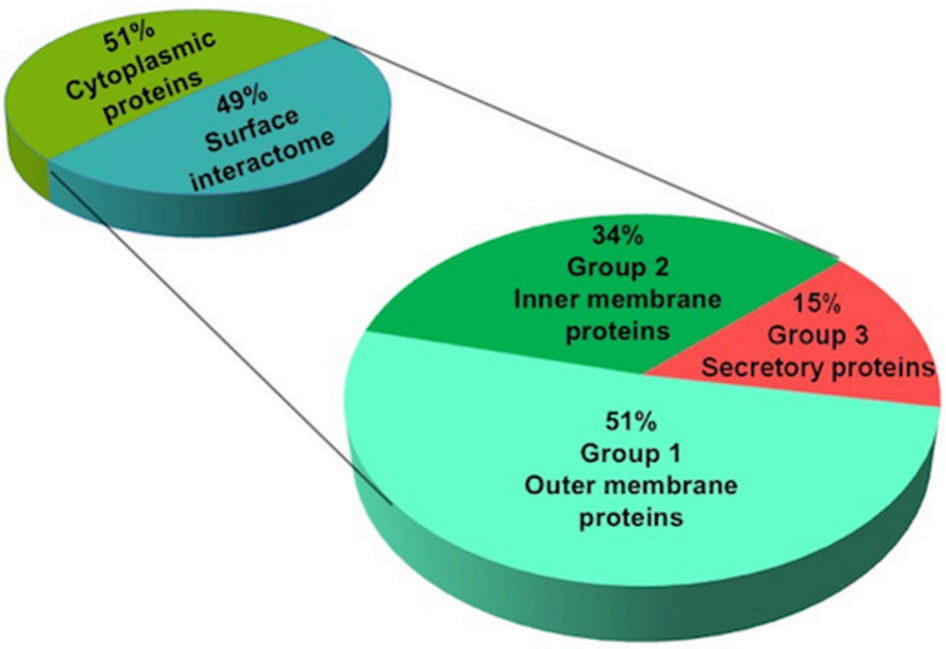
Prediction of subcellular location of identified proteins of *N. meningitidis*. Proteins identified by SWATH-MS were subjected to *in silico* prediction of subcellular location. Psortb and CELLO segregated proteins into two categories (cytoplasmic and surface proteins). Surface proteins (interactome) were further classified into three categories based on their probability of interaction with the host cells: high (containing outer membrane), medium (inner membrane) and low priority candidates (secretory proteins), by applying bioinformatic tools to predict transmembrane domains (TMHMM and HMMtop) and type I and II signal peptides (SignalP and LipoP).

Considering the importance of surface proteins in the crosstalk of pathogen and host cells, we used a systematic pipeline that combines series of bioinformatic tools and literature review to identify surface interactome of *N. meningitidis*. This pipeline was established to shortlist potential ligands by eliminating proteins with less chance to bind hBMECs (e.g., intracellular proteins) and segregating the surface proteins according to their probability to interact with host cells (e.g., outer membrane, inner membrane and secretory proteins). Out of 84 candidates identified by SWATH-MS, 43 proteins were predicted (Cello and Psortb algorithms) as cytoplasmic and were excluded from further analysis due to the low probability of their interaction with the host cells. The remaining 41 proteins were predicted as surface-exposed proteins and were considered as probable surface interactome against hBMECs (Figure 3). For a better selection of ligand candidates, surface interactome was further categorized (SignalP, LipoP, TMHMM, and HMMtop) into three groups according to the type of surface exposure (e.g., outer membrane, inner membrane and secretion). Surface exposure of ligand influences the probability of its contact with the receptor. Group 1. *high priority candidates*–consisted of 21 outer membrane proteins (including six lipoproteins), group 2. *medium priority candidates*–contained 14 inner membrane proteins (including three lipoproteins), and group 3. *low priority candidates*–contained six secretory proteins (Table 1; Figure 3).

**Table 1:** Surface interactome of N. meningitidis against hBMECs identified in our study by SWATH-MS.

**No**.	**Entry**	**Protein name**	**Locus**	**Psortb**	**Cello**	**SignalP**	**LipoP**	**TMHMM**	**HMMTOP**	**Consensus**
**GROUP 1. HIGH PRIORITY CANDIDATES (OUTER MEMBRANE PROTEINS)**										
1	Q7DDI3	Class 5 outer membrane protein	NMB1053	Inner membrane	Outer membrane	Yes	SP1	Outside	Outside	Outer membrane
2	Q9K187	LPS-assembly protein LptD	NMB0280	Outer membrane	Outer membrane	Yes	SP1	Outside	Outside	Outer membrane
3	Q9JXN3	VacJ-related protein	NMB1961	Outer membrane	Outer membrane	Yes	SP1	Outside	Outside	Outer membrane
4	Q9JXK7	Putative adhesin/invasion	NMB1994	Outer membrane	Extracellular	Yes	SP1	Outside	Outside	Outer membrane
5	Q9JS44	Adhesin MafA1	NMB0375	Unknown	Outer membrane	No	SP2	Outside	Outside	Outer membrane
6	Q7DDH4	Putative lipoprotein	NMB1126	Unknown	Extracellular	Yes	SP2	Outside	Outside	Outer membrane
7	Q06379	Lactoferrin-binding protein A (Iron-regulated outer membrane protein A)	NMB1540	Outer membrane	Outer membrane	Yes	SP1	Outside	1 TMD	Outer membrane
8	P0DH58	Major outer membrane protein P.IA	NMB1429	Outer membrane	Outer membrane	Yes	SP1	Outside	1 TMD	Outer membrane
9	Q9K1M2	Putative outer membrane protein	NMB0088	Outer membrane	Outer membrane/Extracellular	Yes	SP1	Outside	1 TMD	Outer membrane
10	P30690	Major outer membrane protein P.IB	NMB2039	Outer membrane	Outer membrane	Yes	SP1	1 TMD	1 TMD	Outer membrane
11	P57026	Outer membrane protein H.8	NMB1533	Outer membrane	Periplasmic	Yes	SP2	Outside	Inside	Outer membrane
12	Q9K0A7	LPS-assembly lipoprotein LptE	NMB0707	Unknown	Cytoplasmic	No	SP2	Outside	Outside	Outer membrane
13	Q9K0K8	Putative adhesin	NMB0586	Outer membrane	Periplasmic	Yes	SP1	Outside	1 TMD	Outer membrane
14	Q9JXB7	Uncharacterized protein	NMB2134	Outer membrane	Outer membrane	No	SP1	1 TMD	1 TMD	Outer membrane
15	Q9K0U9	Transferrin-binding protein 1	NMB0461	Outer membrane	Outer membrane	Yes	SP1	Outside	Outside	Outer membrane
16	Q9K0V0	Transferrin-binding protein 2 (TBP-2)	NMB0460	Unknown	Extracellular	No	SP2	Outside	1 TMD	Outer membrane
17	Q7DDB6	Probable TonB-dependent receptor	NMB1497	Outer membrane	Outer membrane	No	SP1	Outside	Outside	Outer membrane
18	Q7DDJ2	Adhesin	NMB0992	Unknown	Extracellular	Yes	SP1	Outside	Outside	Outer membrane
19	Q9JYP9	Putative lipoprotein NlpD	NMB1483	Unknown	Periplasmic	Yes	SP2	Outside	Outside	Outer membrane
20	Q9JXL6	Adhesion and penetration protein	NMB1985	Outer membrane	Outer membrane	No	SP1	1 TMD	1 TMD	Outer membrane
21	Q9K165	TPR repeat-containing protein	NMB0313	Unknown	Outer membrane	Yes	SP1	Outside	1 TMD	Outer membrane
**GROUP 2. MEDIUM PRIORITY CANDIDATES (INNER MEMBRANE PROTEINS)**										
22	Q9JXD8	Putative adhesin complex protein	NMB2095	Unknown	Periplasmic	Yes	SP1	Outside	1 TMD	Inner membrane
23	Q9JXT1	Lipoprotein	NMB1898	Unknown	Periplasmic	Yes	SP2	Outside	1 TMD	Inner membrane
24	Q9JYG9	Putative lipoprotein	NMB1592	Unknown	Periplasmic	No	SP2	Outside	1 TMD	Inner membrane
25	Q9K0S2	Uncharacterized protein	NMB0506	Unknown	Outer membrane/Periplasmic	Yes	SP1	1 TMD	1 TMD	Inner membrane
26	Q9JZN1	Uncharacterized protein	NMB0979	Inner membrane	Periplasmic/Inner membrane	Yes	SP1	1 TMD	1 TMD	Inner membrane
27	Q9JZR5	Uncharacterized protein	NMB0928	Inner membrane	Periplasmic	No	Cytoplasmic	Outside	1 TMD	Inner membrane
28	Q9JS13	Uncharacterized protein	NMB1147	Cytoplasmic	Periplasmic/Cytoplasmic	No	Cytoplasmic	1 TMD	1 TMD	Inner membrane
29	Q9JY47	Uncharacterized protein	NMB1749	Unknown	Periplasmic	No	Cytoplasmic	1 TMD	1 TMD	Inner membrane
30	Q9K1L2	Uncharacterized protein	NMB0102	Inner membrane	Inner membrane	No	Cytoplasmic	1 TMD	1 TMD	Inner membrane
31	Q9K173	ComEA-related protein	NMB0299	Inner membrane	Periplasmic	No	TMD	1 TMD	1 TMD	Inner membrane
32	Q9JXG2	Uncharacterized protein	NMB2064	Inner membrane	Inner membrane	No	TMD	1 TMD	1 TMD	Inner membrane
33	Q7DDI4	DedA protein	NMB1052	Unknown	Inner membrane	No	TMD	>1 TMD	>1 TMD	Inner membrane
34	Q7DDC1	Uncharacterized protein	NMB1397	Inner membrane	Cytoplasmic	No	TMD	1 TMD	1 TMD	Inner membrane
35	Q7DD63	Outer membrane lipoprotein	NMB1946	Inner membrane	Periplasmic	Yes	SP2	Outside	Outside	Inner membrane
**GROUP 3. LOW PRIORITY CANDIDATES (SECRETORY PROTEINS)**										
36	Q9JYV5	Iron-regulated protein FrpC	NMB1415	Extracellular	Extracellular/	No	Cytoplasmic	Outside	Outside	Secretory protein
37	Q9K0T0	Hemagglutinin/hemolysin-related protein	NMB0493	Outer membrane	Extracellular	No	Cytoplasmic	Outside	Outside	Secretory protein secretory
38	Q9JY23	Hemagglutinin/hemolysin-related protein	NMB1779	Outer membrane	Extracellular	No	SP1	Outside	Outside	Secretory protein
39	Q9JYW0	FrpA/C-related protein	NMB1409	Unknown	Outer membrane	No	Cytoplasmic	Outside	Outside	Secretory protein
40	Q9K0K9	Iron-regulated protein FrpA	NMB0585	Extracellular	Extracellular	No	Cytoplasmic	Outside	Outside	Secretory protein
41	Q9JZ25	Uncharacterized protein	NMB1327	Extracellular	Periplasmic	No	Cytoplasmic	Outside	Outside	Secretory protein

*Categories for the subcellular location were established according to Psortb and Cello. SignalP and LipoP discriminate proteins containing a SP-I type and SP-II type signal peptides, respectively; and proteins containing transmembrane domains (TMD) were predicted by TMHMM and HMMTOP*.

### *In silico* analysis of the *N. meningitidis* surface interactome

The Blast2GO analysis applied to obtain gene ontology annotations showed that 58.6% of proteins (24 out of 41 candidates) or their orthologs are not annotated (neither in UniProt). Among annotated candidates, majority were related to transport (7 out of 41 proteins, included in group 1; Supplementary Data Sheet 2). Five proteins (NMB0280, NMB1497, NMB0461, NMB1126, and NMB1429) out of those seven were annotated as transport proteins, while two proteins are also involved in other functions, such as signal transduction and response to the stress (NMB2039) or cell adhesion and ion binding (NMB0586). A role in cell adhesion was also annotated to adhesin MafA1 protein (NMB0375). Four proteins from group 1 participate in ion binding, namely NMB0586, NMB0585, NMB1533 and the class 5 outer membrane protein NMB1053. The class 5 outer membrane protein and NMB1985 also possesses peptidase activity. Gene ontology terms related to the membrane organization, cellular component assembly, DNA binding, etc. were grouped into the category “miscellaneous” (Supplementary Data Sheet 2). Two proteins (NMB0707 and NMB1483) from group 1 and three proteins (NMB0299, NMB2095, and NMB1946) from group 2 belonged to this category.

As the Blast2GO failed to provide any relevant information for 24 non-annotated proteins, the literature review was performed. Information for four candidates was retrieved. Transferrin-binding protein 2 (NMB0460) and lactoferrin binding protein A (NMB1540) are associated with transport and ion binding, whereas Iron–regulated protein FrpC (NMB1415) and FrpA/C related protein (NMB1409) are reported as ion binding proteins (Supplementary Data Sheet 2).

### Selection of protein candidates for validation of interaction with hBMECs

Following the bioinformatic pipeline, we selected five proteins for the production of their recombinant forms and subsequent validation of interaction with hBMECs. Four proteins were chosen from group 1 (outer membrane proteins) as follows: MafA1 (NMB0375) was selected as its role in adhesion of *N. gonorrhoeae* to the epithelial cells was described in the literature, however, its function in *N. meningitidis* is not studied yet. Similarly, a major outer membrane protein P.IB (NMB2039) is functionally characterized only in *N. gonorrhoeae*. The third candidate, putative adhesin/invasion (neisserial adhesin A-NadA, NMB1994) also possesses affinity to epithelial cells, while the fourth selected candidate (putative lipoprotein-NMB1126) is from lipoprotein family. The fifth protein (membrane lipoprotein, NMB1946) was predicted as an inner membrane protein (group 2) with bioinformatic tools used in this study, however, in the UniProt database it is annotated as an outer membrane protein and is one of possible vaccine candidate (**Table 3** in Supplementary Data Sheet 2).

### Validation of protein-protein interactions and ligand-hBMECs interaction

**Figure 4:**
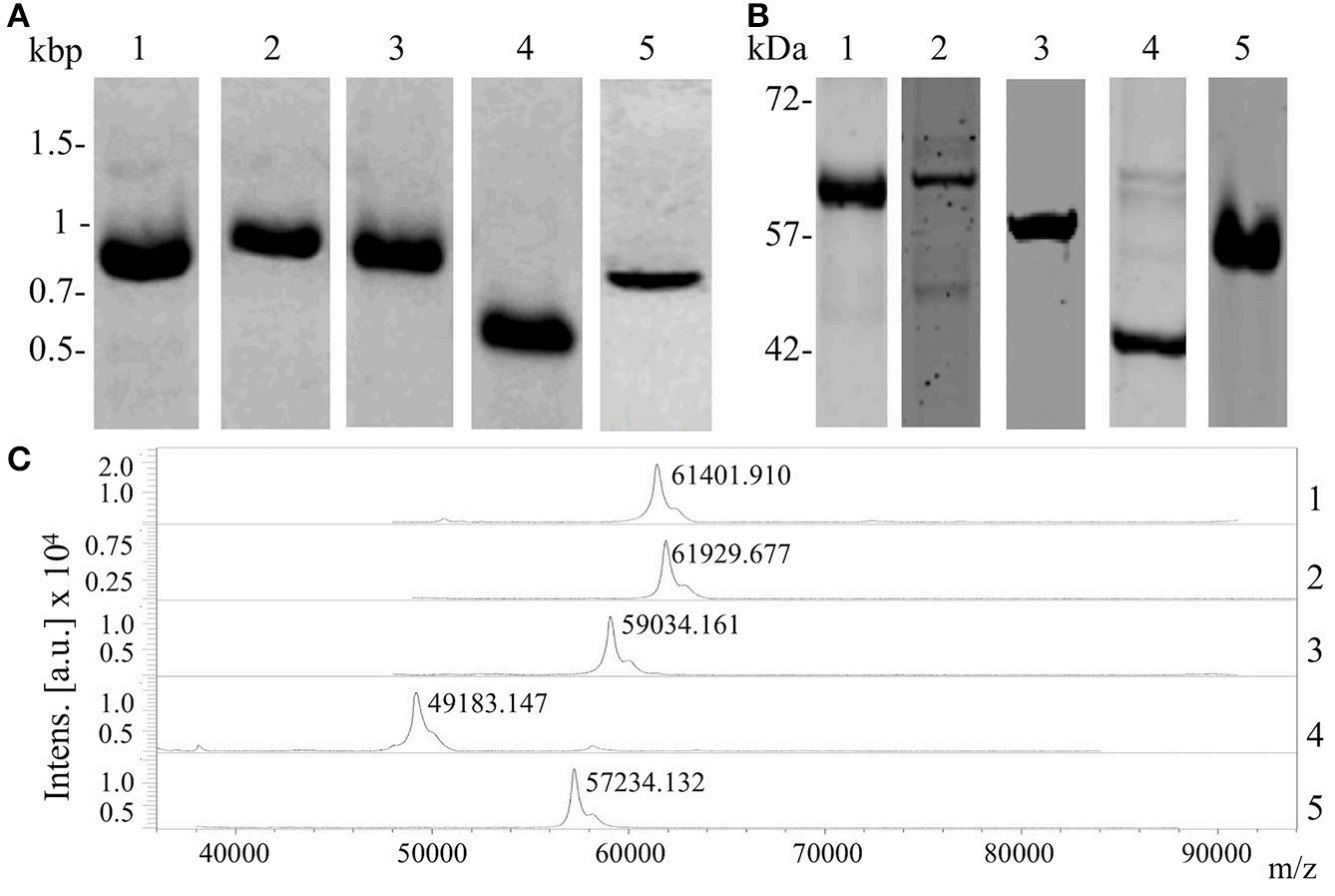
Production of recombinant forms of the selected protein candidates. **(A)** Amplicons of the five gene coding fragments of potential ligands of meningococci resolved on the agarose gel; **(B)** purified recombinant proteins separated with SDS-PAGE; **(C)** molecular mass of recombinant proteins confirmed with MALDI-TOF/MS. Lane 1, adhesin MafA1 (NMB0375); Lane 2, major outer membrane protein P.IB (NMB2039); Lane 3, putative adhesin/invasion (NMB1994); Lane 4, putative lipoprotein (NMB1126); and Lane 5, outer membrane lipoprotein (NMB1946).

**Figure 5:**
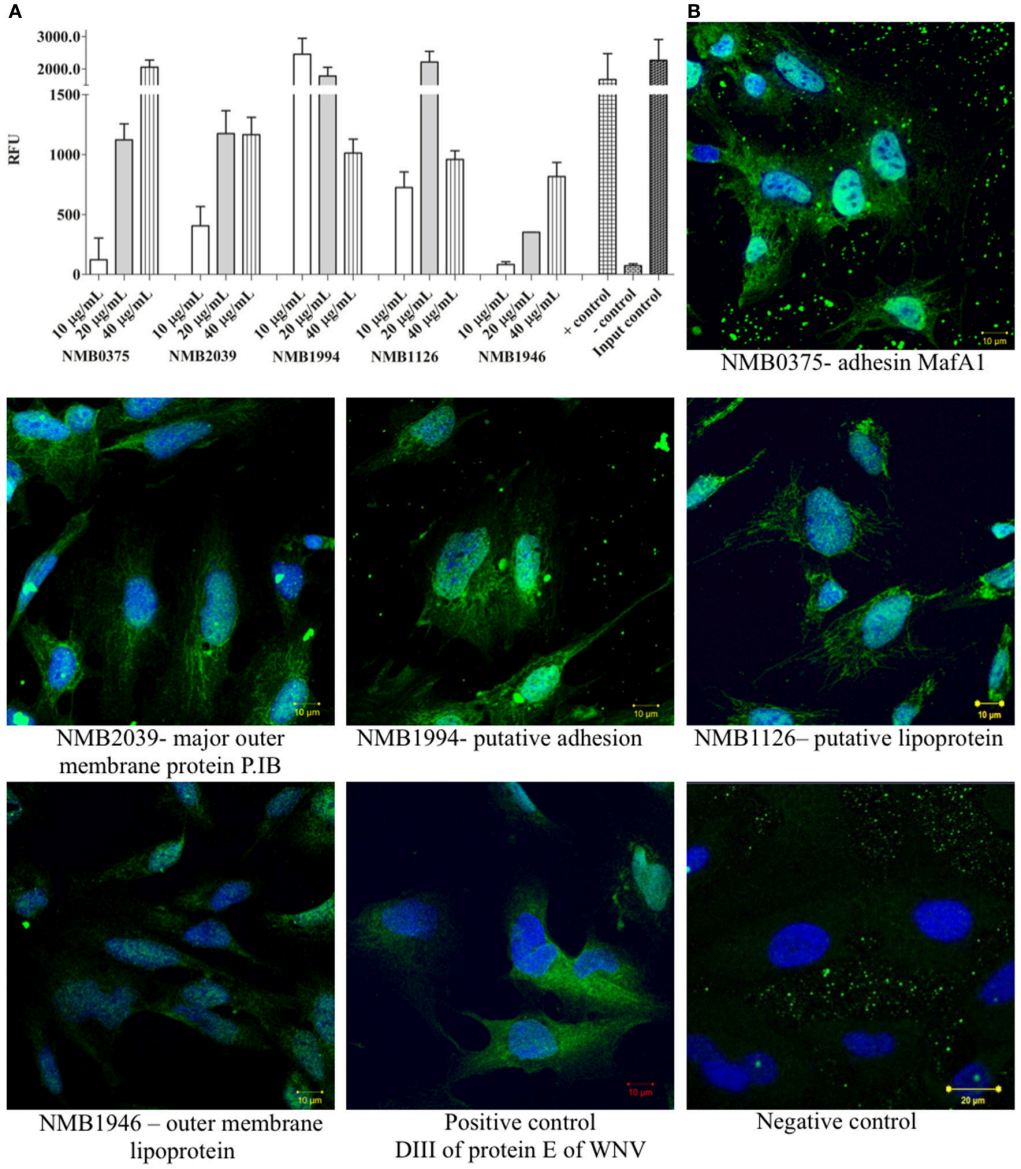
Validation of interaction between selected protein candidates of *N. meningitidis* and cell lysate of hBMECs by semi-quantitative ELISA and immunocytochemistry. **(A)** Semi-quantitative ELISA performed to confirm the interaction between proteins of hBMECs and bacterial ligands (different concentrations). The interaction was detected with HisProbe conjugated with HRP and is shown in Relative Fluorescence Units (RFU). As positive control (+ control)–domain III protein E of West Nile virus was used. Negative control: (– control)–no ligand was added. Input control: recombinant ligand NMB0375 was coated and incubated with HisProbe-HRP and substrate. **(B)** Interaction of selected proteins of *N. meningitidis* with cultured hBMECs. Nuclei are stained with DAPI. Negative control–no recombinant ligand was included in the assay. Scale bars−10 μm. In both panels: NMB0375-adhesin MafA1; NMB2039-major outer membrane protein P.IB; NMB1994-putative adhesin/invasion; NMB1126-putative lipoprotein; NMB1946-outer membrane lipoprotein. Assays were performed in triplicate.

To confirm the interaction between shortlisted ligands and proteins of hBMECs, we produced recombinant forms of ligands (Table 2). Correct insertion of the gene of interest in the transformants, the purity of the recombinant proteins evaluated by SDS-PAGE and molecular masses assessed by MALDI-TOF are shown in Figure 4. The interaction between recombinant ligands and proteins of hBMECs was evaluated by ELISA (Figure 5A). Of note, control experiment without recombinant proteins (negative control, also used for background subtraction, Relative Fluorescence Unit RFU-0.073) confirmed the specificity of the assay. When recombinant ligands were used at 20 μg/mL, binding of putative lipoprotein NMB1126 was the strongest (RFU-2213.3, SD-331.3) followed by putative adhesin/invasion protein NMB1994 (RFU-1776.7, SD-275.4) and NMB2039 major outer membrane protein P.IB (RFU-1175, SD-190.9; Figure 5A). Dose-dependent interaction between hBMECs and recombinant ligands was also assessed, wherein we found that recombinant NMB2039, NMB1994, and NMB1126 were also able to interact with hBMECs at lower concentration (10 μg/mL; Figure 5A). The binding of NMB1946 to hBMECs at 20 μg/mL was the lowest (RFU-235 SD-11.1), however, at 40 μg/mL it appears to bind hBMECs on the same level as other proteins (Figure 5A). RFU for control experiment with domain III of protein E of West Nile virus (positive control) was 1,673.5 with standard deviation 798.8.

**Table 2:** Potential ligands of *N. meningitidis* selected to produce their recombinant form.

**No**.	**Entry UniProt**	**Protein name**	**Locus**	**Amino acids position in recombinant form**	**Mr (kDa)**	
					**Theoretical**	**Observed on SDS-PAGE**
1	Q9JS44	Adhesin MafA1	NMB0375	G30–I307	59.3	≈59
2	P30690	Major outer membrane protein P.IB	NMB2039	A19–F331	62.3	≈60
3	Q9JXK7	Putative adhesin/invasion (NadA)	NMB1994	A135–I328	57.9	≈60
4	Q7DDH4	Putative lipoprotein	NMB1126	E23–G217	48.0	≈49
5	Q7DD63	Outer membrane lipoprotein	NMB1946	Q23–K287	56.0	≈57

We also performed immunocytochemistry to corroborate the interaction between meningococcal ligands and hBMECs. The binding of NMB0375 adhesin MafA and NMB1994 putative adhesin/invasion on endothelial cells was evidently stronger than other three meningococcal ligands (Figure 5B). The binding of domain III of protein E of West Nile virus on endothelial cells was in correlation with its affinity observed in ELISA (Figures 5A,B).

## Discussion

Microbial traversal of the BBB is a prerequisite for CNS infections. Pathogen invading CNS can traverse through the BBB using paracellular or transcellular routes. Initiation of bacterial translocation, via paracellular and transcellular pathways, occurs through the interactions of surface proteins of pathogen and the BBB (Pulzova et al., 2011). These protein-protein interactions (PPIs) provide valuable insight into the molecular networks that unfolds basic principles of pathogen invasion (Nicod et al., 2017). In this study, we attempted to identify the surface interactome of *N. meningitidis* plausibly interacting with hBMECs using high-throughput proteomic methods followed by an integrative bioinformatic analysis. Data from the available scientific publications were brought to draw appropriate interpretations.

Initially, we attempted to label surface proteome of the intact bacterial cells and extract biotin-labeled proteome. However, the labeling efficiency was low and few protein species were recovered (data are not shown). We speculate that capsule present on *N. meningitidis*, hinders labeling of the surface proteins with biotin. Thus, the whole proteome of meningococcus was biotinylated. As a result, it was possible to recover majority of meningococcal proteins that were used to identify interacting candidates with hBMECs (Figure 2A). Biotinylation of the whole cell proteomes or cell surface proteins has been performed in recent proteomic studies (Kay et al., 2009; Hormann et al., 2016; Jimenez-Munguia et al., 2018).

High-throughput approach to identify surface proteins of bacterial pathogens has been well-documented (Vytvytska et al., 2002; Jimenez-Munguia et al., 2018). Among the various mass spectrometry based methods, data-independent acquisition (SWATH-MS) technique is highly sensitive and robust method to accurately identify peptides (Ortea et al., 2016). Due to the high sensitivity of SWATH-MS even the minor interactors (or low abundant proteins) were possible to detect.

In many cases, high-throughput approaches generate a number of probable interactors, from which it is necessary to filter false positive hits. Thus, we have proposed a bioinformatic pipeline to specifically select proteins based on their highest probability of interaction with hBMECs. The implied method of exploiting multiple combinations of freely available bioinformatic tools is noteworthy.

Membrane proteins of Gram-negative bacteria predominantly consist of large, hydrophobic, antiparallel β-barrels that are localized into the membrane with the polar segments protruding at the extracellular region (Jeanteur et al., 1991; von Heijne, 1992). Therefore, it is difficult to resolve these proteins on 2D-PAGE. Applying SWATH-MS we were able to identify 84 proteins. Protein candidates were selected based on their subcellular location through the bioinformatic pipeline described in this work. As the surface exposed proteins are important in the initial stages of paracellular or transcellular pathways, cytoplasmic proteins were excluded from further analysis. The exclusion is also supported in other studies (Krumins and Stotzky, 1983; Maurel et al., 2004; Jimenez-Munguia et al., 2018). Apart from the surface proteins, 43 cytoplasmic proteins were identified that may bind with hBMECs directly or through other surface proteins (Rankl et al., 2006; Jeyachandran et al., 2010).

Thirty-five proteins were predicted as surface-associated in this study (plausible surface interactome). As far as the probability of interaction to the hBMECs is concerned, class 5 outer membrane proteins, ion binding proteins, adhesins and proteins from lipoprotein family are promising candidates in the surface interactome obtained in this study. Opc (NMB1053), a class 5 outer membrane protein was observed in the interactome. Class 5 proteins (e.g., Opc and Opa) and type IV pili (Tfp) enables adhesion of meningococci to the human cells (Virji et al., 1993; Virji, 2009). Earlier it was though that Opc and Opa are hidden under the polysaccharide capsule and are thus inaccessible to their receptors. However, it was later shown that expression of the capsule is diminished during meningococcal infection to allow interaction of surface ligands including Opa and Opc proteins with human cells (Virji et al., 1992, 1993; Dehio et al., 1998). Opa recognizes the members of CEACAM (carcinoembryonic antigen-related cell adhesion molecule) proteins on host cells (Virji et al., 1996), whereas Opc was shown to bind to HSPGs (heparan sulfate proteoglycans), and thus both proteins mediate adhesion and invasion of epithelial cells (de Vries et al., 1998). Although, Pili, Opc and Opa are abundant proteins on the neisserial surface (also referred as major adhesins), other surface proteins expressed in low amount (minor adhesins) might also play a crucial role in the bacterial adhesion and cell invasion. Role of minor adhesins in pathogenesis is predicted earlier (Hill et al., 2010). In this study, we have identified NMB1994-putative adhesin/invasion protein, NMB0586-putative adhesin, NMB2095-putative adhesin complex protein, NMB1985-adhesion/penetration protein and NMB0992-adhesin that may be designated as minor adhesins.

Proteins belonging to the lipoprotein family (e.g., components of the type IV pili) are important virulence factors of *N. meningitidis* as described in Kovacs-Simon et al (Kovacs-Simon et al., 2011). Lipoproteins are also attractive vaccine candidates, either in recombinant forms or in novel outer membrane vesicles (OMV) based vaccines. Putative lipoprotein (NMB1126), adhesin MafA1 (NMB0375), LPS-assembly lipoprotein LptE (NMB0707), outer membrane protein H.8 (NMB1533), putative lipoprotein NlpD (NMB1483) and lipoprotein (NMB1898) were among the identified candidates with lipid moiety in our study. Ion binding proteins are another group of proteins, considered as an important pathogenicity factor for meningococcal disease (Cornelissen and Sparling, 1994; Cornelissen, 2003). In the repertoire of surface interactome we found several ion binding candidates *viz*. transferrin-binding proteins Tbp1 NMB0461 and Tbp2 NMB0460 (mediating iron acquisition in the host), iron-regulated protein FrpA (NMB1409), iron-regulated protein FrpC (NMB1415), lactoferrin-binding protein A (NMB1540) and transferrin-binding protein 1 (NMB046; Table 1). Lactoferrin-binding protein A (LbpA) participates in recruitment of lactoferrin and is an important virulence factor of the meningococci (Pettersson et al., 1994). It is noteworthy that, lactoferrin is able to cross the BBB during an acute phase of inflammation and serves as a resource of iron during the invasion (Huettinger et al., 1998; Pettersson et al., 1998). This underlines the importance of LbpA in the process of neuroinvasion.

After segregation of protein candidates according to their subcellular location, bioinformatic pipeline described in this study was applied to collect relevant biological data for each shortlisted protein. Blast2GO provided gene ontology annotations for 17 protein interactors. The GO analysis has assigned cell adhesion, transport and ion binding functions for putative adhesin (NMB0586). Another putative adhesin/invasion protein (NMB1994), had 100% similarity with neisserial adhesin A (NadA), which has been reported to interact with epithelial cells (Capecchi et al., 2005). Interaction of NadA with hBMECs confirmed in this study (Figure 5) further adds to its significance in pathogenicity of *N. meningitidis*. Unfortunately, Blast2GO failed to retrieve annotations for 41.4% proteins, while literature review (Supplementary Data Sheet 2) provided information only on four non-annotated proteins [NMB0460, NMB1540, NMB1415 and NMB1409 that are associated with ion binding (Thompson and Sparling, 1993b; Thompson et al., 1993a)]. These non-annotated proteins needs further exploration to determine their biological function in pathogenesis.

Among five potential ligands selected for validation of interaction with hBMECs four proteins (NMB0375, NMB2039, NMB1994, and NMB1126) were selected from outer membrane proteins (group 1) because their surface exposure may facilitate contact and interaction with hBMECs. Relatively less is known about MafA1 (NMB0375) in *N. meningitidis*, however, it is reported that MafA1 of *N. gonorrhoeae* interacts with gangliotriosylceramide (GgO_3_) and gangliotetraosylceramide (GgO_4_) expressed on human endocervical cells (Paruchuri et al., 1990). Major outer membrane protein P.IB (NMB2039), another selected candidate belongs to the group of porins, abundantly localized on the outer membrane of pathogenic *Neisseria* (Wetzler, 2010). The interaction of P.IB protein with hBMECs and its subsequent effect on cell signaling has not yet been reviewed. However, it was found that P.IB of *N. gonorrhoeae* (PorB_IA_) binds to the scavenger receptor on endothelial cells (SREC-I), which leads to the bacterial uptake into endothelial or epithelial cells (Rechner et al., 2007). It is plausible that P.IB of *N. meningitidis* identified in the present study could bind to SREC-I of hBMECs. Third selected candidate, putative adhesin/invasion (neisserial adhesin A-NadA, NMB1994) is described to interact strongly with epithelial cells (Capecchi et al., 2005) but not with hBMECs. The fourth candidate selected for validation was the putative lipoprotein (NMB1126), a member of the lipoprotein family. NMB1946 (the fifth selected candidate) was reported to be a potential vaccine candidate against N. *meningitidis* (Pizza et al., 2000; Neumoin et al., 2011). NMB1946 and its homolog in N. *gonorrhoeae* were found upregulated in the presence of blood and a knockout mutant was found to be less competitive than the wild type strain (Echenique-Rivera et al., 2011). Interaction of above mentioned proteins with hBMECs was validated with both ELISA and immunocytochemistry (Figure 5).

To conclude, the proposed workflow is suitable for high-throughput screening of interacting proteins. This approach allowed us to identify interactome of the *N. meningitidis* against hBMECs for the selection of potential ligands. Furthermore, we have experimentally confirmed the interaction of five recombinant ligands with hBMECs. This systematic combination of proteomics and bioinformatic tools can potentially be applied to different microorganisms in deciphering their interactome at varying environmental conditions.

## Author contributions

MB conceived the project. Experiments were designed by EK and MB. Biotinylation was performed by IJ-M and EK. Sequencing was carried out by KB and ZT. Human BMECs were grown by PeM, KB, and EK. LP, EK, and ZT produced recombinant proteins. AK performed MS analysis. PaM and PeM contributed in immunocytochemistry. The bioinformatic pipeline was proposed by EK and IJ-M. Data were analyzed by IJ-M and MB. EK, IJ-M, and MB wrote the manuscript. All authors read and approved the final manuscript.

### Conflict of interest statement

The authors declare that the research was conducted in the absence of any commercial or financial relationships that could be construed as a potential conflict of interest.
