# Supplementary material for: Deciphering the Interactome of Neisseria meningitidis With Human Brain Microvascular Endothelial Cells
Source: Front Microbiol. 2018 Sep 26;9:2294. doi: 10.3389/fmicb.2018.02294 (PMC6168680; doi:10.3389/fmicb.2018.02294)
Supplement: Supplementary file 1 [file Data_Sheet_1.PDF]

Supplementary material data sheet 1

Amino acid and nucleotide sequences of recombinant proteins, Primer locations

In gray are highlighted the amino acid and nucleotide sequences included to make recombinant form of proteins. Sequences highlighted with yellow color are primer sequences.

Protein name: Adhesin MafA1

Uniprot accession number: Q9JS44

aa sequence: 313

MKTLLLLIPLVLTACGTLTGIPAHGGGKRFAVEQELVAASSRAAVKEMDLSALKGRK
AALYVSVMGDQGSGNISGGRYSIDALIRGGYHNNPESATQYSYPAYDTTATTKSDAL
SSVTTSTSLLNAPAAALTKNSGRKGERSAGLSVNGTGDYRNETLLANPRDVSFLTNLI
QTVFYLRGIEVVPPEYADTDVFVTVDVFGTVRSRTELHLYNAETLKAQTKLEYFAVD
RDSRKLLITPKTAAYESQYQEQYALWTGPYKVSKTVKASDRLMVDFSDITPYGDTTA
QNRPDFKQNNGKKPDVGNEVIRRRKGG

Genbank accession number: AE002098.2: ntc378274 to ntc377333 (Length of the gene 942 bp)

ATGAAAACCCTGCTCCTCCTCATCCCCCTCGTCCTCACAGCCTGCGGCACACTGAC
CGGCATACCCGCCCACGGCGGCGGCAAACGCTTTGCCGTCGAACAAGAACTCGTC
GCCGCATCGTCCCGCGCCGCCGTCAAAGAAATGGATTTGTCCGCCCTAAAAGGAC
GCAAAGCCGCCCTTTACGTCTCCGTTATGGGCGACCAAGGTTCGGGCAACATAAG
CGGCGGACGCTACTCTATCGACGCACTGATACGCGGCGGCTACCACAACAACCCC
GAAAGTGCCACCCAATACAGCTACCCCGCCTACGACACTACCGCCACCACCAAAT
CCGACGCGCTCTCCAGCGTAACCACTTCCACATCGCTTTTGAACGCCCCCGCCGC
CGCCCTGACGAAAAACAGCGGACGCAAAGGCGAACGCTCCGCCGGACTGTCCGT
CAACGGCACGGGCGACTACCGCAACGAAACCCTGCTCGCCAACCCCCGCGACGT
TTCCTTCCTGACCAACCTCATCCAAACCGTCTTCTACCTGCGCGGCATCGAAGTCG
TACCGCCCGAATACGCCGACACCGACGTATTCGTAACCGTCGACGTATTCGGCAC
CGTCCGCAGCCGTACCGAACTGCACCTCTACAACGCCGAAACCCTTAAAGCCCAA
ACCAAGCTCGAATATTTCGCCGTTGACCGCGACAGCCGGAAACTGCTGATTACCC
CTAAAACCGCCGCCTACGAATCCCAATACCAAGAACAATACGCCCTTTGGACCGG
CCCTTACAAAGTCAGCAAAACCGTCAAAGCCTCAGACCGCCTGATGGTCGATTTC
TCCGACATTACCCCCTACGGCGACACAACCGCCCAAAACCGTCCCGACTTCAAAC
AAAACAACGGTAAAAAACCCGATGTCGGCAACGAAGTCATCCGCCGCCGCAAAG
GAGGATAA

Protein name: Major outer membrane protein P.IB

Uniprot accession number: P30690
aa sequence: 331
MKKSLIALTLAALPVAAMADVTLYGTIKAGVETSRSVFHQNGQVTEVTTATGIVDLG
SKIGFKGQEDLGNGLKAIWQVEQKASIAGTDSGWGNRQSFIGLKGGFGKLRVGRLNS
VLKDTGDINPWDSKSDYLGVNKIAEPEARLISVRYDSPEFAGLSGSVQYALNDNAGR
HNSESYHAGFNYKNGGFFVQYGGAYKRHHQVQEGLNIEKYQIHRLVSGYDNDALY
ASVAVQQQDAKLTDASNSHNSQTEVAATLAYRFGNVTPRVSYAHGFKGLVDDADIG
NEYDQVVVGAEYDFSKRTSALVSAGWLQEGKGENKFVATAGGVGLRHKF

Genbank accession number: AE002098.2*: nt2157529 to nt2158524 (Length of the gene 996 bp)
ATGAAAAAATCCCTGATTGCCCTGACTTTGGCAGCCCTTCCTGTTGCAGCAATGG
CTGACGTTACCCTGTACGGCACCATCAAAGCCGGCGTAGAAACTTCCCGCTCTGT
ATTTCACCAGAACGGCCAAGTTACTGAAGTTACAACCGCTACCGGCATCGTTGAT
TTGGGTTCGAAAATCGGCTTCAAAGGCCAAGAAGACCTCGGTAACGGCCTGAAA
GCCATTTGGCAGGTTGAGCAAAAAGCATCTATCGCCGGTACTGACTCCGGTTGGG
GCAACCGCCAATCCTTCATCGGCTTGAAAGGCGGCTTCGGTAAATTGCGCGTCGG
TCGTTTGAACAGCGTCCTGAAAGACACCGGCGACATCAATCCTTGGGATAGCAAA
AGCGACTATTTGGGTGTAAACAAAATTGCCGAACCCGAGGCACGCCTCATTTCCG
TACGCTACGATTCTCCCGAATTTGCCGGCCTCAGCGGCAGCGTACAATACGCGCT
TAACGACAATGCAGGCAGACATAACAGCGAATCTTACCACGCCGGCTTCAACTAC
AAAAACGGTGGCTTCTTCGTGCAATATGGCGGTGCCTATAAAAGACATCATCAAG
TGCAAGAGGGCTTGAATATTGAGAAATACCAGATTCACCGTTTGGTCAGCGGTTA
CGACAATGATGCCCTGTACGCTTCCGTAGCCGTACAGCAACAAGACGCGAAACTG
ACTGATGCTTCCAATTCGCACAACTCTCAAACCGAAGTTGCCGCTACCTTGGCAT
ACCGCTTCGGCAACGTAACGCCCCGAGTTTCTTACGCCCACGGCTTCAAAGGTTT
GGTTGATGATGCAGACATAGGCAACGAATACGACCAAGTGGTTGTCGGTGCGGA
ATACGACTTCTCCAAACGCACTTCTGCCTTGGTTTCTGCCGGTTGGTTGCAAGAAG
GCAAAGGCGAAAACAAATTCGTAGCGACTGCCGGCGGTGTCGGTCTGCGCCACA
AATTCTAA

Protein name: Putative adhesin/invasion

Uniprot accession number: Q9JXK7
aa sequence: 364
MSMKHFPSKVLTTAILATFCSGALAATSDDDVKKAATVAIVAAYNNGQEINGFKAG
ETIYDIGEDGTITQKDATAADVEADDFKGLGLKKVVTNLTKTVNENKQNVDAKVKA
AESEIEKLTTKLADTDAALADTDAALDETTNALNKLGENITTFAEETKTNIVKIDEKL
EAVADTVDKHAEAFNDIADSLDETNTKADEAVKTANEAKQTAEETKQNVDAKVKA
AETAAGKAEAAAGTANTAADKAEAVAAKVTDIKADIATNKADIAKNSARIDSLDKN
VANLRKETRQGLAEQAALSGLFQPYNVGRFNVTAAVGGYKSESAVAIGTGFRFTENF
AAKAGVAVGTSSGSSAAYHVGVNYEW

Genbank accession number: AE002098.2*: nt2100383 to nt2101477 (Length of the gene 1095 bp)
ATGAGCATGAAACACTTTCCATCCAAAGTACTGACCACAGCCATCCTTGCCACTT
TCTGTAGCGGCGCACTGGCAGCCACAAGCGACGACGATGTTAAAAAAGCTGCCA
CTGTGGCCATTGTTGCTGCCTACAACAATGGCCAAGAAATCAACGGTTTCAAAGC
TGGAGAGACCATCTACGACATTGGTGAAGACGGCACAATTACCCAAAAAGACGC
AACTGCAGCCGATGTTGAAGCCGACGACTTTAAAGGTCTGGGTCTGAAAAAAGTC
GTGACTAACCTGACCAAAACCGTCAATGAAAACAAACAAAACGTCGATGCCAAA
GTAAAAGCTGCAGAATCTGAAATAGAAAAGTTAACAACCAAGTTAGCAGACACT
GATGCCGCTTTAGCAGATACTGATGCCGCTCTGGATGAAACCACCAACGCCTTGA
ATAAATTGGGAGAAAATATAACGACATTTGCTGAAGAGACTAAGACAAATATCG
TAAAAATTGATGAAAAATTAGAAGCCGTGGCTGATACCGTCGACAAGCATGCCG
AAGCATTCAACGATATCGCCGATTCATTGGATGAAACCAACACTAAGGCAGACG
AAGCCGTCAAAACCGCCAATGAAGCCAAACAGACGGCCGAAGAAACCAAACAA
AACGTCGATGCCAAAGTAAAAGCTGCAGAAACTGCAGCAGGCAAAGCCGAAGCT
GCCGCTGGCACAGCTAATACTGCAGCCGACAAGGCCGAAGCTGTCGCTGCAAAA
GTTACCGACATCAAAGCTGATATCGCTACGAACAAAGCTGATATTGCTAAAAACT
CAGCACGCATCGACAGCTTGGACAAAAACGTAGCTAATCTGCGCAAAGAAACCC
GCCAAGGCCTTGCAGAACAAGCCGCGCTCTCCGGCCTGTTCCAACCTTACAACGT
GGGTCGGTTCAATGTAACGGCTGCAGTCGGCGGCTACAAATCCGAATCGGCAGTC
GCCATCGGTACCGGCTTCCGCTTTACCGAAAACTTTGCCGCCAAAGCAGGCGTGG
CAGTCGGCACTTCGTCCGGTTCTTCCGCAGCCTACCATGTCGGCGTCAATTACGA
GTGGTAA

Protein name: Putative lipoprotein NMB1126/NMB1164

Uniprot accession number: Q7DDH4
aa sequence: 223
MKTVSTAVVLAAAAVSLTGCATESSRSLEVEKVASYNTQYHGVRTPISVGTFDNRSS
FQKGIFSDGEDRLGSQAKTILVTHLQQTNRFNVLNRTNLNALKQESGISGKAHNLKG
ADYVVTGDVTEFGRRDVGDHQLFGILGRGKSQIAYAKVALNIVNVNTSEIVYSAQGA
GEYALSNREIIGFGGTSGYDATLNGKVLDLAIREAVNSLVQAVDNGAWQPNR

Genbank accession number: AE002098.2*: ntc1137355 to ntc1136684 (Length of the gene 672 bp)
ATGAAAACCGTTTCCACCGCCGTTGTCCTTGCCGCCGCTGCCGTTTCACTGACCGG
CTGTGCGACCGAATCCTCACGCAGTCTCGAGGTAGAGAAAGTCGCCTCCTACAAT
ACGCAATATCACGGTGTTCGTACCCCGATTTCCGTCGGAACATTCGACAACCGCT
CCAGCTTCCAAAAAGGCATTTTCTCCGACGGGGAAGACCGTTTGGGCAGCCAGGC
AAAAACCATTCTAGTAACGCACCTGCAACAGACCAACCGCTTCAACGTACTGAAC
CGCACCAATTTGAACGCATTAAAACAGGAATCCGGCATTTCCGGCAAAGCGCATA
ACCTGAAAGGCGCAGATTATGTCGTTACCGGCGATGTAACCGAATTCGGACGCAG
AGATGTCGGCGATCATCAGCTCTTCGGCATTTTGGGTCGCGGCAAATCGCAAATC
GCCTATGCAAAAGTGGCTCTGAATATCGTCAACGTCAATACTTCCGAAATCGTCT
ATTCCGCACAGGGCGCGGGCGAATACGCACTTTCCAACCGTGAAATCATCGGTTT
CGGCGGCACTTCCGGCTACGATGCGACTTTGAACGGCAAAGTTTTAGACTTGGCA
ATCCGCGAAGCCGTCAACAGCCTGGTTCAGGCTGTTGACAACGGCGCATGGCAAC
CCAACCGTTAA

Protein name: Outer membrane lipoprotein

Uniprot accession number: Q7DD63
aa sequence: 287
MKTFFKTLSAAALALILAACGGQKDSAPAASASAAADNGAAKKEIVFGTTVGDFGD
MVKEQIQAELEKKGYTVKLVEFTDYVRPNLALAEGELDINVFQHKPYLDDFKKEHN
LDITEVFQVPTAPLGLYPGKLKSLEEVKDGSTVSAPNDPSNFARVLVMLDELGWIKL
KDGINPLTASKADIAENLKNIKIVELEAAQLPRSRADVDFAVVNGNYAISSGMKLTEA
LFQEPSFAYVNWSAVKTADKDSQWLKDVTEAYNSDAFKAYAHKRFEGYKSPAAWN
EGAAK

Genbank accession number: AE002098.2*: ntc2041945 to ntc2041082 (Length of the gene 864 bp)
ATGAAAACCTTCTTCAAAACCCTTTCCGCCGCCGCACTCGCGCTCATCCTCGCCGC
CTGCGGCGGTCAAAAAGACAGCGCGCCCGCCGCATCCGCTTCTGCCGCCGCCGACAACG
GCGCGGCGAAAAAAGAAATCGTCTTCGGCACGACCGTCGGCGACTTCGGCGATATGGTCAAAGAA
CAAATCCAAGCCGAGCTGGAGAAAAAAGGCTACACCGTCAAACTGGTCGAGTTTACCGACTATGT
ACGCCCGAATCTGGCATTGGCTGAGGGCGAGTTGGACATCAACGTCTTCCAACACAAACCCTATCT
TGACGACTTCAAAAAAGAACACAATCTGGACATCACCGAAGTCTTCCAAGTGCCGACCGCGCCTTT
GGGACTGTACCCGGGCAAGCTGAAATCGCTGGAAGAAGTCAAAGACGGCAGCACCGTATCCGCGC
CCAACGACCCGTCCAACTTCGCCCGCGTCTTGGTGATGCTCGACGAACTGGGTTGGATCAAACTCA
AAGACGGCATCAATCCGTTGACCGCATCCAAAGCGGACATCGCCGAGAACCTGAAAAACATCAAA
ATCGTCGAGCTTGAAGCCGCGCAACTGCCGCGTAGCCGCGCCGACGTGGATTTTGCCGTCGTCAAC
GGCAACTACGCCATAAGCAGCGGCATGAAGCTGACCGAAGCCCTGTTCCAAGAACCGAGCTTTGC
CTATGTCAACTGGTCTGCCGTCAAAACCGCCGACAAAGACAGCCAATGGCTTAAAGACGTAACCG
AGGCCTATAACTCCGACGCGTTCAAAGCCTACGCGCACAAACGCTTCGAGGGCTACAAATCCCCTG
CCGCATGGAATGAAGGCGCAGCCAAATAA
