# Supplementary material for: Deciphering the Interactome of Neisseria meningitidis With Human Brain Microvascular Endothelial Cells
Source: Front Microbiol. 2018 Sep 26;9:2294. doi: 10.3389/fmicb.2018.02294 (PMC6168680; doi:10.3389/fmicb.2018.02294)
Supplement: Supplementary file 2 [file Data_Sheet_2.PDF]

Supplementary material data sheet 2

Table 1. Primers used to produce recombinant forms of the ligands

No.	Protein/ (Gene)	Sequence used to design primers	Primer	Sequence (5´- 3´)	Amplicon lenght (bp)
1	Adhesin MafA1 (*mafA1*)	AE002098.2*: ntc378274 to ntc377333	*mafA1*- sense<br>*mafA1*- antisense	TATGGATCCGGCAAACGCTTTGCCGTCGAAC<br>TTTGGTACCGATGACTTCGTTGCCGACATC	843
2	Major outer membrane protein P.IB *(porB)*	AE002098.2*: nt2157529 to nt2158524	*porB*- sense<br>*porB*- antisense	TATGGATCCGCTGACGTTACCCTGTACGGC<br>TTTGTCGACGAATTTGTGGCGCAGACCGAC	939
3	Putative adhesin/invasion (*NMB1994*)	AE002098.2*: nt2100383 to nt2101477	*NMB1994*- sense<br>*NMB1994*- antisense	TATGGATCCGCTGCCACTGTGGCCATTGTT<br>TTTGGTACCGATGGCGACTGCCGATTCGGA	884
4	Putative lipoprotein (*NMB1126*)	AE002098.2*: ntc1137355 to ntc1136684	*NMB1126*- sense<br>*NMB1126*- antisense	TATGGATCCGAATCCTCACGCAGTCTCGAG<br>TTTGTCGACGCCGTTGTCAACAGCCTGAAC	585
5	Outer membrane lipoprotein (*NMB1946*)	AE002098.2*: ntc2041945 to ntc2041082	*NMB1946*- sense<br>*NMB1946*- antisense	TATGGATCCCAAAAAGACAGCGCGCCCGCC<br>TTTGTCGACTTTGGCTGCGCCTTCATTCCATG C	795

*shows Genbank accession number followed by nucleotide positions spanning the gene. Restriction sites are depicted with underlined nucleotides. GGATCC- *BamH*I, GGTACC – *Kpn*I and GTCGAC - *Sal*I

Table 2. Primers used for insertom PCR	
Primer	Sequence (5´- 3´)
UA Insertom F1	CGCATCACCATCACCATCACG
UA Insertom R1	ACCAAATTGGGACAACACCAGTG

Table 3. Ontology and Literature review based categorization of the surface interactome

No.	Entry	Protein name	Locus	Ontology[a]							Literature[b]					
				Cell adhesion	Peptidase activity	Transport	Ion binding	Signal trans-duction	Response to stress	Misc.	Cell adhesion	Trans-port	Ion binding	VC	Misc.	References
**Group 1. High priority candidates (outer membrane proteins)**																
1	Q9JS44	Adhesin MafA1	NMB0375	X							X					(1)
2	P57026	Outer membrane protein H.8	NMB1533				X									
3	Q9K0K8	Putative adhesin	NMB0586	X		X	X					X	X			(2, 3)
4	Q7DDI3	Class 5 outer membrane protein	NMB1053		X		X			X	X					(4, 5)
5	Q9JXL6	Adhesion and penetration protein	NMB1985		X											
6	P30690	Major outer membrane protein P.IB	NMB2039			X*		X	X	X	X	X	X	X		(6-9)
7	Q9K187	LPS-assembly protein LptD	NMB0280			X									X	(10)
8	Q7DDB6	Probable TonB-dependent receptor	NMB1497			X						X				(2)
9	Q9K0U9	Transferrin-binding protein 1	NMB0461			X						X	X			(11, 12)
10	Q7DDH4	Putative lipoprotein	NMB1126			X						X				(13-15)
11	P0DH58	Major outer membrane protein P.IA	NMB1429			X*							X	X		(6, 16)
12	Q9K0V0	Transferrin-binding protein 2 (TBP-2)	NMB0460									X	X			(11, 12)
13	Q06379	Lactoferrin-binding protein A (Iron-regulated outer membrane protein A)	NMB1540									X	X			(17)
14	Q9K0A7	LPS-assembly lipoprotein LptE	NMB0707							X					X	(10)
15	Q9JYP9	Putative lipoprotein NlpD	NMB1483							X						
16	Q9K165	TPR repeat-containing protein	NMB0313													
17	Q9JXN3	VacJ-related protein	NMB1961													
18	Q9JXK7	Putative adhesin/invasion	NMB1994													
19	Q9K1M2	Putative outer membrane protein	NMB0088													
20	Q9JXB7	Uncharacterized protein	NMB2134													
21	Q7DDJ2	Adhesin	NMB0992													
**Group 2. Medium priority candidates (inner membrane proteins)**																
22	Q9K173	ComEA-related protein	NMB0299						X	X						
23	Q9JXD8	Putative adhesin complex protein	NMB2095							X						
24	Q7DD63	Outer membrane lipoprotein	NMB1946							X				X	X	(18, 19)
25	Q9JXT1	Lipoprotein	NMB1898													
26	Q9JYG9	Putative lipoprotein	NMB1592													

27	Q9K0S2	Uncharacterized protein	NMB0506													
28	Q9JZN1	Uncharacterized protein	NMB0979													
29	Q9JZR5	Uncharacterized protein	NMB0928													
30	Q9JS13	Uncharacterized protein	NMB1147													
31	Q9JY47	Uncharacterized protein	NMB1749													
32	Q9K1L2	Uncharacterized protein	NMB0102													
33	Q9JXG2	Uncharacterized protein	NMB2064													
34	Q7DDI4	DedA protein	NMB1052													
35	Q7DDC1	Uncharacterized protein	NMB1397													
**Group 3. Low priority candidates (secretory proteins)**																
36	Q9K0K9	Iron-regulated protein FrpA	NMB0585				X						X			(20, 21)
37	Q9JYV5	Iron-regulated protein FrpC	NMB1415										X			(20, 21)
38	Q9JYW0	FrpA/C-related protein	NMB1409										X			(20, 21)
39	Q9K0T0	Hemagglutinin/hemolysin-related protein	NMB0493													
40	Q9JY23	Hemagglutinin/hemolysin-related protein	NMB1779													
41	Q9JZ25	Uncharacterized protein	NMB1327													

[a] Categories of the functional analysis were established according to BLAST2GO predictions.

[b] Categories assigned based on literature review; VC: vaccine candidate.

Empty cells- not assigned function or not described in literature.

* transmembrane transport activity included in transport.

Misc. - miscellaneous category includes functions such as membrane organization, cellular component assembly, cell division, nucleotidyltransferase activity, oxidoreductase activity, DNA binding, DNA metabolic process or cellular nitrogen compound metabolic process.

References

1. Paruchuri DK, Seifert HS, Ajioka RS, Karlsson KA, So M. Identification and characterization of a *Neisseria gonorrhoeae* gene encoding a glycolipid-binding adhesin. *Proceedings of the National Academy of Sciences of the United States of America* (1990) 87(1):333-7. Epub 1990/01/01. PubMed PMID: 2153292; PubMed Central PMCID: PMC53257.

2. Stork M, Bos MP, Jongerius I, de Kok N, Schilders I, Weynants VE, et al. An outer membrane receptor of *Neisseria meningitidis* involved in zinc acquisition with vaccine potential. *PLoS pathogens* (2010) 6:e1000969. Epub 2010/07/10. doi: 10.1371/journal.ppat.1000969. PubMed PMID: 20617164; PubMed Central PMCID: PMC2895646.

3. Pawlik MC, Hubert K, Joseph B, Claus H, Schoen C, Vogel U. The zinc-responsive regulon of *Neisseria meningitidis* comprises 17 genes under control of a Zur element. *Journal of bacteriology* (2012) 194(23):6594-603. Epub 2012/10/09. doi: 10.1128/JB.01091-12. PubMed PMID: 23043002; PubMed Central PMCID: PMC3497534.

4. Virji M, Makepeace K, Ferguson DJ, Watt SM. Carcinoembryonic antigens (CD66) on epithelial cells and neutrophils are receptors for Opa proteins of pathogenic neisseriae. *Molecular microbiology* (1996) 22(5):941-50. Epub 1996/12/01. PubMed PMID: 8971715.

5. de Vries FP, Cole R, Dankert J, Frosch M, van Putten JP. *Neisseria meningitidis* producing the Opc adhesin binds epithelial cell proteoglycan receptors. *Molecular microbiology* (1998) 27(6):1203-12. Epub 1998/05/07. PubMed PMID: 9570405.

6. Song J, Minetti CA, Blake MS, Colombini M. Successful recovery of the normal electrophysiological properties of PorB (class 3) porin from *Neisseria meningitidis* after expression in Escherichia coli and renaturation. *Biochimica et biophysica acta* (1998) 1370(2):289-98. Epub 1998/05/16. PubMed PMID: 9545584.

7. Minetti CA, Tai JY, Blake MS, Pullen JK, Liang SM, Remeta DP. Structural and functional characterization of a recombinant PorB class 2 protein from *Neisseria meningitidis*. Conformational stability and porin activity. *The Journal of biological chemistry* (1997) 272(16):10710-20. Epub 1997/04/18. PubMed PMID: 9099721.

8. Massari P, Ho Y, Wetzler LM. *Neisseria meningitidis* porin PorB interacts with mitochondria and protects cells from apoptosis. *Proceedings of the National Academy of Sciences of the United States of America* (2000) 97(16):9070-5. Epub 2000/08/02. PubMed PMID: 10922061; PubMed Central PMCID: PMC16823.

9. Kozjak-Pavlovic V, Dian-Lothrop EA, Meinecke M, Kepp O, Ross K, Rajalingam K, et al. Bacterial porin disrupts mitochondrial membrane potential and sensitizes host cells to apoptosis. *PLoS pathogens* (2009) 5(10):e1000629. Epub 2009/10/24. doi: 10.1371/journal.ppat.1000629. PubMed PMID: 19851451; PubMed Central PMCID: PMC2759283.

10. Bos MP, Tommassen J. The LptD chaperone LptE is not directly involved in lipopolysaccharide transport in *Neisseria meningitidis*. *The Journal of biological chemistry* (2011) 286(33):28688-96. Epub 2011/06/28. doi: 10.1074/jbc.M111.239673. PubMed PMID: 21705335; PubMed Central PMCID: PMC3190676.

11. Cornelissen CN. Transferrin-iron uptake by Gram-negative bacteria. *Frontiers in bioscience : a journal and virtual library* (2003) 8:d836-47. Epub 2003/04/18. PubMed PMID: 12700102.

12. Cornelissen CN, Sparling PF. Iron piracy: acquisition of transferrin-bound iron by bacterial pathogens. *Molecular microbiology* (1994) 14(5):843-50. Epub 1994/12/01. PubMed PMID: 7715446.

13. Finney M, Vaughan T, Taylor S, Hudson MJ, Pratt C, Wheeler JX, et al. Characterization of the key antigenic components and pre-clinical immune responses to a meningococcal disease vaccine based on *Neisseria lactamica* outer membrane vesicles. *Human vaccines* (2008) 4(1):23-30. Epub 2007/10/09. PubMed PMID: 17921703.

14. Loferer H, Hammar M, Normark S. Availability of the fibre subunit CsgA and the nucleator protein CsgB during assembly of fibronectin-binding curli is limited by the intracellular concentration of the novel lipoprotein CsgG. *Molecular microbiology* (1997) 26(1):11-23. Epub 1998/01/31. PubMed PMID: 9383186.

15. Robinson LS, Ashman EM, Hultgren SJ, Chapman MR. Secretion of curli fibre subunits is mediated by the outer membrane-localized CsgG protein. *Molecular microbiology* (2006) 59(3):870-81. Epub 2006/01/20. doi: 10.1111/j.1365-2958.2005.04997.x. PubMed PMID: 16420357; PubMed Central PMCID: PMC2838483.

16. Tommassen J, Vermeij P, Struyve M, Benz R, Poolman JT. Isolation of *Neisseria meningitidis* mutants deficient in class 1 (porA) and class 3 (porB) outer membrane proteins. *Infection and immunity* (1990) 58(5):1355-9. Epub 1990/05/01. PubMed PMID: 2157669; PubMed Central PMCID: PMC258632.

17. Pettersson A, Klarenbeek V, van Deurzen J, Poolman JT, Tommassen J. Molecular characterization of the structural gene for the lactoferrin receptor of the meningococcal strain H44/76. *Microbial pathogenesis* (1994) 17(6):395-408. Epub 1994/12/01. doi: 10.1006/mpat.1994.1085. PubMed PMID: 7752881.

18. Pizza M, Scarlato V, Masignani V, Giuliani MM, Arico B, Comanducci M, et al. Identification of vaccine candidates against serogroup B meningococcus by whole-genome sequencing. *Science* (2000) 287(5459):1816-20. Epub 2000/03/10. PubMed PMID: 10710308.

19. Echenique-Rivera H, Muzzi A, Del Tordello E, Seib KL, Francois P, Rappuoli R, et al. Transcriptome analysis of *Neisseria meningitidis* in human whole blood and mutagenesis studies identify virulence factors involved in blood survival. *PLoS pathogens* (2011) 7(5):e1002027. Epub 2011/05/19. doi: 10.1371/journal.ppat.1002027. PubMed PMID: 21589640; PubMed Central PMCID: PMC3088726.

20. Thompson SA, Wang LL, Sparling PF. Cloning and nucleotide sequence of frpC, a second gene from *Neisseria meningitidis* encoding a protein similar to RTX cytotoxins. *Molecular microbiology* (1993) 9(1):85-96. Epub 1993/07/01. PubMed PMID: 8412674.

21. Thompson SA, Sparling PF. The RTX cytotoxin-related FrpA protein of *Neisseria meningitidis* is secreted extracellularly by meningococci and by HlyBD+ *Escherichia coli*. *Infection and immunity* (1993) 61(7):2906-11. Epub 1993/07/01. PubMed PMID: 8514394; PubMed Central PMCID: PMC280938.
