# Supplementary material for: Deciphering the Interactome of Neisseria meningitidis With Human Brain Microvascular Endothelial Cells
Source: Front Microbiol. 2018 Sep 26;9:2294. doi: 10.3389/fmicb.2018.02294 (PMC6168680; doi:10.3389/fmicb.2018.02294)
Supplement: Supplementary file 3 [file Data_Sheet_3.PDF]

Supplementary material data sheet 3

Identification of potential ligands of *N. meningitidis* interacting with human BMEC by SWATH-MS

No.	Scores	#Peptides	SC [%]	RMS90 [ppm]	Entry	Entry name	Protein names	Gene names	*Organism*
1	2377.4 (M:2377.4)	19	99.3	12.54	Q7DDJ2	tr|Q7DDJ2|Q7DDJ2_NEIMB	Adhesin	hsf NMB0992	*Neisseria meningitidis serogroup B (strain MC58)*
2	1779.3 (M:1779.3)	18	87.8	4.05	Q9JS44	sp|Q9JS44|MAFA_NEIMB	Adhesin MafA	mafA1 NMB0375; mafA2 NMB0652	*Neisseria meningitidis serogroup B (strain MC58)*
3	1255.6 (M:1255.6)	16	30.4	12.17	Q9JXL6	tr|Q9JXL6|Q9JXL6_NEIMB	Adhesion and penetration protein	hap NMB1985	*Neisseria meningitidis serogroup B (strain MC58)*
4	903.2 (M:903.2)	11	86.4	6.76	P30690	sp|P30690|OMPB1_NEIMB	Major outer membrane protein P.IB (PIB) (Protein IB) (Class 3 protein) (Porin)	porB NMB2039	*Neisseria meningitidis*
5	727.0 (M:727.0)	11	34.1	9.69	Q9K0E1	tr|Q9K0E1|Q9K0E1_NEIMB	AmpD protein	ampD NMB0668	*Neisseria meningitidis serogroup B (strain MC58)*
6	690.4 (M:690.4)	9	38.1	11.75	Q9K145	tr|Q9K145|Q9K145_NEIMB	BolA/YrbA family protein	NMB0344	*Neisseria meningitidis serogroup B (strain MC58)*
7	683.4 (M:683.4)	11	33.7	6.64	Q7DDI3	tr|Q7DDI3|Q7DDI3_NEIMB	Class 5 outer membrane protein	opc NMB1053	*Neisseria meningitidis serogroup B (strain MC58)*
8	639.5 (M:639.5)	13	20.8	14.66	Q9K173	tr|Q9K173|Q9K173_NEIMB	ComEA-related protein	NMB0299	*Neisseria meningitidis serogroup B (strain MC58)*
9	610.7 (M:610.7)	9	18.0	7.69	Q7DDH4	sp|Q7DDH4|Y1126_NEIMB	Putative lipoprotein NMB1126/NMB1164	NMB1126; NMB1164	*Neisseria meningitidis*
10	586.3 (M:586.3)	6	12.9	12.24	Q7DDI4	tr|Q7DDI4|Q7DDI4_NEIMB	DedA protein	dedA NMB1052	*Neisseria meningitidis serogroup B (strain MC58)*
11	547.7 (M:547.7)	7	21.7	5.23	Q7DDG9	tr|Q7DDG9|Q7DDG9_NEIMB	Ferredoxin, 2Fe-2S type	fdx-1 fdx-2 NMB1134 NMB1172	*Neisseria meningitidis serogroup B (strain MC58)*
12	537.6 (M:537.6)	7	45.7	7.49	Q9JXL8	sp|Q9JXL8|LUXS_NEIMB	S-ribosylhomocysteine lyase (EC 4.4.1.21) (AI-2 synthesis protein) (Autoinducer-2 production protein LuxS)	luxS NMB1981	*Neisseria meningitidis*
13	511.0 (M:511.0)	8	46.6	10.81	Q9JYW0	tr|Q9JYW0|Q9JYW0_NEIMB	FrpA/C-related protein	NMB1409	*Neisseria meningitidis serogroup B (strain MC58)*
14	473.8 (M:473.8)	6	17.1	13.57	Q9JXW8	sp|Q9JXW8|CARB_NEIMB	Carbamoyl-phosphate synthase large chain (EC 6.3.5.5) (Carbamoyl-phosphate synthetase ammonia chain)	carB NMB1855	*Neisseria meningitidis*

15	472.5 (M:472.5)	7	18.4	7.81	Q9K0T0	tr|Q9K0T0|Q9K0T0_NEIMB	Hemagglutinin/hemolysin-related protein	NMB0493	*Neisseria meningitidis serogroup B (strain MC58)*
16	461.1 (M:461.1)	4	36.4	10.83	Q9JY23	tr|Q9JY23|Q9JY23_NEIMB	Hemagglutinin/hemolysin-related protein	NMB1779	*Neisseria meningitidis serogroup B (strain MC58)*
17	454.7 (M:454.7)	7	13.1	9.12	Q9K0K9	sp|Q9K0K9|FRPA_NEIMB	Iron-regulated protein FrpA	frpA NMB0585	*Neisseria meningitidis serogroup B (strain MC58)*
18	449.2 (M:449.2)	8	56.5	9.35	Q9JYV5	sp|Q9JYV5|FRPC_NEIMB	Iron-regulated protein FrpC	frpC NMB1415	*Neisseria meningitidis serogroup B (strain MC58)*
19	440.3 (M:440.3)	6	13.6	13.61	Q9K0A8	tr|Q9K0A8|Q9K0A8_NEIMB	Laccase domain protein	NMB0706	*Neisseria meningitidis serogroup B (strain MC58)*
20	391.4 (M:391.4)	5	12.9	7.03	Q06379	sp|Q06379|LBPA_NEIMB	Lactoferrin-binding protein A (Iron-regulated outer membrane protein A)	lbpA iroA NMB1540	*Neisseria meningitidis serogroup B (strain MC58)*
21	354.4 (M:354.4)	7	40.5	3.38	Q9K0I2	sp|Q9K0I2|PPSA_NEIMB	Phosphoenolpyruvate synthase (PEP synthase) (EC 2.7.9.2) (Pyruvate, water dikinase)	ppsA NMB0618	*Neisseria meningitidis*
22	351.7 (M:351.7)	6	12.7	9.07	Q9K0N4	sp|Q9K0N4|DNAK_NEIMB	Chaperone protein DnaK (HSP70) (Heat shock 70 kDa protein) (Heat shock protein 70)	dnaK NMB0554	*Neisseria meningitidis*
23	339.6 (M:339.6)	3	12.1	8.99	Q7DD63	tr|Q7DD63|Q7DD63_NEIMB	Lipoprotein	NMB1946	*Neisseria meningitidis serogroup B (strain MC58)*
24	334.8 (M:334.8)	7	10.2	8.62	Q9JXT1	tr|Q9JXT1|Q9JXT1_NEIMB	Lipoprotein	mlp NMB1898	*Neisseria meningitidis serogroup B (strain MC58)*
25	334.1 (M:334.1)	3	24.8	3.60	Q9K0A7	tr|Q9K0A7|Q9K0A7_NEIMB	LPS-assembly lipoprotein LptE	lptE NMB0707	*Neisseria meningitidis serogroup B (strain MC58)*
26	333.2 (M:333.2)	4	19.8	10.30	Q9K187	sp|Q9K187|LPTD_NEIMB	LPS-assembly protein LptD	lptD imp ostA NMB0280	*Neisseria meningitidis serogroup B (strain MC58)*
27	332.9 (M:332.9)	4	12.7	10.24	P0DH58	sp|P0DH58|OMPA_NEIMB	Major outer membrane protein P.IA (PIA) (Protein IA) (Class 1 protein)	porA NMB1429	*Neisseria meningitidis serogroup B (strain MC58)*
28	328.5 (M:328.5)	4	22.8	6.18	P57026	sp|P57026|H8_NEIMB	Outer membrane protein H.8	NMB1533	*Neisseria meningitidis serogroup B (strain MC58)*
29	315.8 (M:315.8)	4	27.6	12.36	Q9JZZ3	tr|Q9JZZ3|Q9JZZ3_NEIMB	PmbA protein	pmbA NMB0839	*Neisseria meningitidis serogroup B (strain MC58)*
30	315.2 (M:315.2)	5	16.4	10.14	Q7DDK0	tr|Q7DDK0|Q7DDK0_NEIMB	Citrate synthase	gltA NMB0954	*Neisseria meningitidis*
31	294.8 (M:294.8)	2	8.7	13.78	Q7DDK2	tr|Q7DDK2|Q7DDK2_NEIMB	Succinate dehydrogenase iron-sulfur subunit (EC 1.3.5.1)	sdhB NMB0951	*Neisseria meningitidis*
32	276.9 (M:276.9)	4	21.7	7.62	Q7DDS5	tr|Q7DDS5|Q7DDS5_NEIMB	Catalase (EC 1.11.1.6)	kat NMB0216	*Neisseria meningitidis*

33	276.1 (M:276.1)	6	22.1	7.34	Q9JX95	tr|Q9JX95|Q9JX95_NEIMB	Glyceraldehyde-3-phosphate dehydrogenase (EC 1.2.1.-)	gapA-2 NMB2159	*Neisseria meningitidis*
34	275.9 (M:275.9)	4	24.6	11.61	Q9JXK6	tr|Q9JXK6|Q9JXK6_NEIMB	Nitrogen regulatory protein P-II	glnB NMB1995	*Neisseria meningitidis*
35	275.4 (M:275.4)	3	30.7	4.23	Q9JXV5	tr|Q9JXV5|Q9JXV5_NEIMB	Fructose-bisphosphate aldolase (EC 4.1.2.13)	cbbA NMB1869	*Neisseria meningitidis*
36	270.8 (M:270.8)	3	16.3	12.75	Q9JY94	tr|Q9JY94|Q9JY94_NEIMB	D-lactate dehydrogenase (EC 1.1.1.28)	ldhA NMB1685	*Neisseria meningitidis*
37	256.5 (M:256.5)	4	17.9	3.52	Q9JYQ7	tr|Q9JYQ7|Q9JYQ7_NEIMB	Aminotransferase, class I (EC 2.6.1.-)	NMB1473	*Neisseria meningitidis*
38	256.2 (M:256.2)	2	22.9	12.82	Q9JZ28	tr|Q9JZ28|Q9JZ28_NEIMB	Thioredoxin reductase (EC 1.8.1.9)	trxB NMB1324	*Neisseria meningitidis*
39	250.7 (M:250.7)	4	15.0	5.54	Q7DDB6	sp|Q7DDB6|Y1497_NEIMB	Probable TonB-dependent receptor NMB1497	NMB1497	*Neisseria meningitidis serogroup B (strain MC58)*
40	243.2 (M:243.2)	3	24.6	6.39	Q9K0K8	tr|Q9K0K8|Q9K0K8_NEIMB	Putative adhesin	NMB0586	*Neisseria meningitidis serogroup B (strain MC58)*
41	243.2 (M:243.2)	4	7.8	12.57	Q9JXD8	tr|Q9JXD8|Q9JXD8_NEIMB	Putative adhesin complex protein	NMB2095	*Neisseria meningitidis serogroup B (strain MC58)*
42	240.7 (M:240.7)	4	34.0	9.01	Q9JXK7	tr|Q9JXK7|Q9JXK7_NEIMB	Putative adhesin/invasin	NMB1994	*Neisseria meningitidis serogroup B (strain MC58)*
43	240.2 (M:240.2)	3	16.1	6.37	P63700	sp|P63700|BFRB_NEIMB	Putative bacterioferritin B (BFR B)	bfrB NMB1206	*Neisseria meningitidis serogroup B (strain MC58)*
44	236.1 (M:236.1)	3	16.3	14.16	Q9JYG9	tr|Q9JYG9|Q9JYG9_NEIMB	Putative lipoprotein	NMB1592	*Neisseria meningitidis serogroup B (strain MC58)*
45	232.8 (M:232.8)	5	10.2	15.24	Q9JYP9	tr|Q9JYP9|Q9JYP9_NEIMB	Putative lipoprotein NlpD	NMB1483	*Neisseria meningitidis serogroup B (strain MC58)*
46	229.5 (M:229.5)	6	24.6	6.40	Q9K1M2	sp|Q9K1M2|Y088_NEIMB	Putative outer membrane protein NMB0088	NMB0088	*Neisseria meningitidis serogroup B (strain MC58)*
47	224.6 (M:224.6)	5	30.9	14.97	Q9JY49	tr|Q9JY49|Q9JY49_NEIMB	Putative tspB protein	NMB1747	*Neisseria meningitidis serogroup B (strain MC58)*
48	1255.6 (M:1255.6)	16	30.4	12.17	Q9JXG4	tr|Q9JXG4|Q9JXG4_NEIMB	ThiF protein	thiF NMB2062	*Neisseria meningitidis serogroup B (strain MC58)*
49	903.2 (M:903.2)	11	86.4	6.76	Q9JXG0	tr|Q9JXG0|Q9JXG0_NEIMB	TldD protein	tldD NMB2066	*Neisseria meningitidis serogroup B (strain MC58)*
50	727.0 (M:727.0)	11	34.1	9.69	Q9K165	sp|Q9K165|Y0313_NEIMB	TPR repeat-containing protein NMB0313	NMB0313	*Neisseria meningitidis serogroup B (strain MC58)*

51	690.4 (M:690.4)	9	38.1	11.75	Q9K0U9	sp|Q9K0U9|TBP1_NEIMB	Transferrin-binding protein 1	tbp1 NMB0461	*Neisseria meningitidis serogroup B (strain MC58)*
52	683.4 (M:683.4)	11	33.7	6.64	Q9K0V0	sp|Q9K0V0|TBPB_NEIMB	Transferrin-binding protein 2 (TBP-2)	tbpB tbp2 NMB0460	*Neisseria meningitidis serogroup B (strain MC58)*
53	639.5 (M:639.5)	13	20.8	14.66	Q9JRV5	tr|Q9JRV5|Q9JRV5_NEIMB	Uncharacterized protein	NMB1128 NMB1166	*Neisseria meningitidis serogroup B (strain MC58)*
54	610.7 (M:610.7)	9	18.0	7.69	Q7DDG2	tr|Q7DDG2|Q7DDG2_NEIMB	Uncharacterized protein	NMB1142 NMB1180	*Neisseria meningitidis serogroup B (strain MC58)*
55	586.3 (M:586.3)	6	12.9	12.24	Q9JS13	tr|Q9JS13|Q9JS13_NEIMB	Uncharacterized protein	NMB1147 NMB1185	*Neisseria meningitidis serogroup B (strain MC58)*
56	547.7 (M:547.7)	7	21.7	5.23	Q9JZW7	tr|Q9JZW7|Q9JZW7_NEIMB	Uncharacterized protein	NMB0868	*Neisseria meningitidis serogroup B (strain MC58)*
57	275.4 (M:275.4)	3	30.7	4.23	Q9JYI1	tr|Q9JYI1|Q9JYI1_NEIMB	Uncharacterized protein	NMB1575	*Neisseria meningitidis serogroup B (strain MC58)*
58	270.8 (M:270.8)	3	16.3	12.75	Q9JZC6	tr|Q9JZC6|Q9JZC6_NEIMB	Uncharacterized protein	NMB1114	*Neisseria meningitidis serogroup B (strain MC58)*
59	256.5 (M:256.5)	4	17.9	3.52	Q9K1P8	tr|Q9K1P8|Q9K1P8_NEIMB	Uncharacterized protein	NMB0032	*Neisseria meningitidis serogroup B (strain MC58)*
60	256.2 (M:256.2)	2	22.9	12.82	Q9JXA2	tr|Q9JXA2|Q9JXA2_NEIMB	Uncharacterized protein	NMB2152	*Neisseria meningitidis serogroup B (strain MC58)*
61	250.7 (M:250.7)	4	15.0	5.54	Q9K019	tr|Q9K019|Q9K019_NEIMB	Uncharacterized protein	NMB0808	*Neisseria meningitidis serogroup B (strain MC58)*
62	243.2 (M:243.2)	3	24.6	6.39	Q9K1C5	tr|Q9K1C5|Q9K1C5_NEIMB	Uncharacterized protein	NMB0237	*Neisseria meningitidis serogroup B (strain MC58)*
63	243.2 (M:243.2)	4	7.8	12.57	Q9JZK4	tr|Q9JZK4|Q9JZK4_NEIMB	Uncharacterized protein	NMB1013	*Neisseria meningitidis serogroup B (strain MC58)*
64	240.7 (M:240.7)	4	34.0	9.01	Q9K0S2	tr|Q9K0S2|Q9K0S2_NEIMB	Uncharacterized protein	NMB0506	*Neisseria meningitidis serogroup B (strain MC58)*
65	240.2 (M:240.2)	3	16.1	6.37	Q9JZN1	tr|Q9JZN1|Q9JZN1_NEIMB	Uncharacterized protein	NMB0979	*Neisseria meningitidis serogroup B (strain MC58)*
66	240.2 (M:240.2)	3	16.1	6.37	Q9K1L2	tr|Q9K1L2|Q9K1L2_NEIMB	Uncharacterized protein	NMB0102	*Neisseria meningitidis serogroup B (strain MC58)*
67	236.1 (M:236.1)	3	16.3	14.16	Q9JYK1	tr|Q9JYK1|Q9JYK1_NEIMB	Uncharacterized protein	NMB1547	*Neisseria meningitidis serogroup B (strain MC58)*
68	232.8 (M:232.8)	5	10.2	15.24	Q9K196	tr|Q9K196|Q9K196_NEIMB	Uncharacterized protein	NMB0271	*Neisseria meningitidis serogroup B (strain MC58)*

69	229.5 (M:229.5)	6	24.6	6.40	Q9JXG2	tr|Q9JXG2|Q9JXG2_NEIMB	Uncharacterized protein	NMB2064	*Neisseria meningitidis serogroup B (strain MC58)*
70	224.6 (M:224.6)	5	30.9	14.97	Q9JY47	tr|Q9JY47|Q9JY47_NEIMB	Uncharacterized protein	NMB1749	*Neisseria meningitidis serogroup B (strain MC58)*
71	1255.6 (M:1255.6)	16	30.4	12.17	Q9JZK8	tr|Q9JZK8|Q9JZK8_NEIMB	Uncharacterized protein	NMB1008	*Neisseria meningitidis serogroup B (strain MC58)*
72	903.2 (M:903.2)	11	86.4	6.76	Q9JYD1	tr|Q9JYD1|Q9JYD1_NEIMB	Uncharacterized protein	NMB1644	*Neisseria meningitidis serogroup B (strain MC58)*
73	727.0 (M:727.0)	11	34.1	9.69	Q9JY11	tr|Q9JY11|Q9JY11_NEIMB	Uncharacterized protein	NMB1796	*Neisseria meningitidis serogroup B (strain MC58)*
74	690.4 (M:690.4)	9	38.1	11.75	Q9K1G4	tr|Q9K1G4|Q9K1G4_NEIMB	Uncharacterized protein	NMB0189	*Neisseria meningitidis serogroup B (strain MC58)*
75	683.4 (M:683.4)	11	33.7	6.64	Q9JZT5	tr|Q9JZT5|Q9JZT5_NEIMB	Uncharacterized protein	NMB0906	*Neisseria meningitidis serogroup B (strain MC58)*
76	639.5 (M:639.5)	13	20.8	14.66	Q9K028	tr|Q9K028|Q9K028_NEIMB	Uncharacterized protein	NMB0797	*Neisseria meningitidis serogroup B (strain MC58)*
77	610.7 (M:610.7)	9	18.0	7.69	Q7DDC1	tr|Q7DDC1|Q7DDC1_NEIMB	Uncharacterized protein	NMB1397	*Neisseria meningitidis serogroup B (strain MC58)*
78	586.3 (M:586.3)	6	12.9	12.24	Q9JYA9	tr|Q9JYA9|Q9JYA9_NEIMB	Uncharacterized protein	NMB1667	*Neisseria meningitidis serogroup B (strain MC58)*
79	240.2 (M:240.2)	3	16.1	6.37	Q9K0Z5	tr|Q9K0Z5|Q9K0Z5_NEIMB	Uncharacterized protein	NMB0406	*Neisseria meningitidis serogroup B (strain MC58)*
80	236.1 (M:236.1)	3	16.3	14.16	Q9JXB7	tr|Q9JXB7|Q9JXB7_NEIMB	Uncharacterized protein	NMB2134	*Neisseria meningitidis serogroup B (strain MC58)*
81	232.8 (M:232.8)	5	10.2	15.24	Q9JZR5	sp|Q9JZR5|Y928_NEIMB	Uncharacterized protein NMB0928	NMB0928	*Neisseria meningitidis serogroup B (strain MC58)*
82	229.5 (M:229.5)	6	24.6	6.40	Q9JZ25	sp|Q9JZ25|Y1327_NEIMB	Uncharacterized protein NMB1327	NMB1327	*Neisseria meningitidis serogroup B (strain MC58)*
83	224.6 (M:224.6)	5	30.9	14.97	Q9JZ20	sp|Q9JZ20|Y1333_NEIMB	Uncharacterized protein NMB1333	NMB1333	*Neisseria meningitidis serogroup B (strain MC58)*
84	1255.6 (M:1255.6)	16	30.4	12.17	Q9JXN3	tr|Q9JXN3|Q9JXN3_NEIMB	VacJ-related protein	NMB1961	*Neisseria meningitidis serogroup B (strain MC58)*
